# Perfluorochemical‐facilitated plasminogen activator delivery to the airways: A novel treatment for inhalational smoke‐induced acute lung injury

**DOI:** 10.1002/ctm2.26

**Published:** 2020-04-30

**Authors:** Marla R. Wolfson, Perenlei Enkhbaatar, Satoshi Fukuda, Christina L. Nelson, Robert O. Williams, Soraya Hengsawas Surasarang, Sawittree Sahakijpijarn, Gennaro Calendo, Andrey A. Komissarov, Galina Florova, Krishna Sarva, Steven I. Idell, Thomas H. Shaffer

**Affiliations:** ^1^ Department of Thoracic Medicine & Surgery, Physiology & Pediatrics, and Temple Lung Center Lewis Katz School of Medicine at Temple University Philadelphia Pennsylvania USA; ^2^ Department of Anesthesiology The University of Texas Medical Branch Galveston Texas USA; ^3^ College of Pharmacy The University of Texas at Austin Austin Texas USA; ^4^ Cellular and Molecular Biology and the Texas Lung Institute The University of Texas Health Science Center at Tyler Tyler Texas USA; ^5^ Biomedical Research School of Medicine Temple and Thomas Jefferson Schools of Medicine Alfred I. duPont Hospital for Children Wilmington Delaware USA

**Keywords:** airway casts, inhalational acute lung injury, perfluorochemicals, plasminogen activators

## Abstract

**Background:**

Effective clinical management of airway clot and fibrinous cast formation of severe inhalational smoke‐induced acute lung injury (ISALI) is lacking. Aerosolized delivery of tissue plasminogen activator (tPA) is confounded by airway bleeding; single‐chain urokinase plasminogen activator (scuPA) moderated this adverse effect and supported transient improvement in gas exchange and lung mechanics. However, neither aerosolized plasminogen activator (PA) yielded durable improvements in physiologic responses or reduction in cast burden. Here, we hypothesized that perfluorochemical (PFC) liquids would facilitate PA distribution and sustain improvements in physiologic outcomes in ISALI.

**Methods:**

Spontaneously breathing adult sheep (n = 36) received anesthesia and analgesia and were instrumented, exposed to cotton smoke inhalation, and supported by mechanical ventilation for 48 h. Groups (n = 6/group) were studied without supplemental treatment, or, starting 4 h post injury, they received intratracheal low volume (8 mL) PFC liquid alone or a dose range of tPA/PFC or scuPA/PFC suspensions (4 or 8 mg in 8 mL PFC) every 8 h. Outcomes were evaluated by sequential measurements of cardiopulmonary parameters, lung histomorphology, and biochemical analyses of bronchoalveolar lavage fluid.

**Results:**

Dose‐response and PA‐type comparisons of outcomes demonstrated sustained superiority with low‐volume PFC suspensions of scuPA over tPA or PFC alone, favoring the highest dose of scuPA/PFC suspension over lower doses, without airway bleeding.

**Conclusions:**

We propose that this improved profile over previously reported aerosolized delivery is likely related to improved dose distribution. Sustained salutary responses to scuPA/PFC suspension delivery in this translational model are encouraging and support the possibility that the observed outcomes could be of clinical importance.

## 1 BACKGROUND

Evidence from translational animal models substantiated by clinical experience has demonstrated that inhaled smoke exposure damages the airway epithelial lining.^1^, ^2^, ^3^ This is followed by leakage of plasma substrates into the airways with airway clotting and fibrinous cast formation, resulting in inhalational smoke‐induced acute lung injury (ISALI).^4^ Standard supportive intensive care management of ISALI typically utilizes conventional mechanical ventilation (pressure/volume) support, oxygen therapy, bronchodilators, heparin, mucolytics, and fluid management.^3^, ^5^ Although anticoagulant and plasminogen activator (PA) strategies have been tried in preclinical studies, effective clinical therapy to manage ISALI is still problematic.^3^, ^4^, ^6^ We inferred that smoke‐induced local impairment of fibrinolytic activity contributes to deposition and persistence of airway casts. We used a well‐established sheep model of cotton ISALI and investigated aerosolized delivery of tissue plasminogen activator (tPA) or single‐chain urokinase plasminogen activator (scuPA) in saline.^7^ We found that high‐dose nebulized tPA delivery was confounded by airway bleeding complications, while scuPA was well tolerated and supported transient improvement in gas exchange and lung mechanics. The better response to scuPA appeared to relate to its relative resistance to plasminogen activator inhibitor (PAI‐1) and ability to form bioactive complexes amenable to low‐grade release of uPA for up to 24 h.^7^, ^8^ However, neither of the aerosolized interventions achieved durable reduction in cast burden or improvements in physiologic responses in this severe lung injury model.^7^

Over the last three decades, a number of preclinical investigations have been conducted in which perfluorochemical (PFC)‐facilitated pulmonary drug was tested.^9^, ^10^, ^11^, ^12^, ^13^, ^14^, ^15^, ^16^ Given that the distribution of inhaled aerosolized agents is complicated by the pathology they are targeted to treat, we posited that PFC liquids facilitate the distribution of the PAs and improve outcomes in ISALI. The benefits of PFC‐facilitated drug delivery include that these liquids are inert, can be homogenously distributed in the lung because of low surface tension, mitigate barotrauma, support gas exchange, improve lung mechanics, and recruit lung volume in animal models of ALI.^17^, ^18^, ^19^ Additionally, PFC liquids do not alter the biologics to be delivered and have been shown to decrease inflammatory and oxidative stress responses in the airways and lungs.^18^, ^20^ These effects could be of advantage in ISALI. Furthermore, there is a translational predicate for this approach as specific PFC compounds have been used in infants, children, and adults with severe respiratory distress.^21^, ^22^, ^23^, ^24^

Building on our experience with PFC biomedical applications, development of PFC/drug suspensions for intrapulmonary drug administration, as well as extensive experience with the sheep model of ISALI,^3^, ^4^, ^7^, ^25^, ^26^, ^27^ we also hypothesized that PFC delivery of PA agents mitigates the airway cast formation in ISALI. On the basis of our previous findings using the same sheep model of ISALI, we inferred that better delivery of scuPA using PFC would more effectively improve outcomes by virtue of resistance to PAI‐1 and durable release of PA activity.^7^, ^8^ With more effective local PA delivery, we postulated that cast management would be improved and result in sustained physiologic improvement for the 48‐h study period. In this study, we sought to compare outcomes between PFC alone versus no intervention and PFC alone versus treatments with PFC suspensions of tPA or scuPA and, further, to determine dose‐dependent differences between the PAs delivered intratracheally beginning at 4 h after injury and then every 8 h‐48 h.

## 2 MATERIALS AND METHODS

### 2.1 Sheep model of ISALI

**FIGURE 1:**
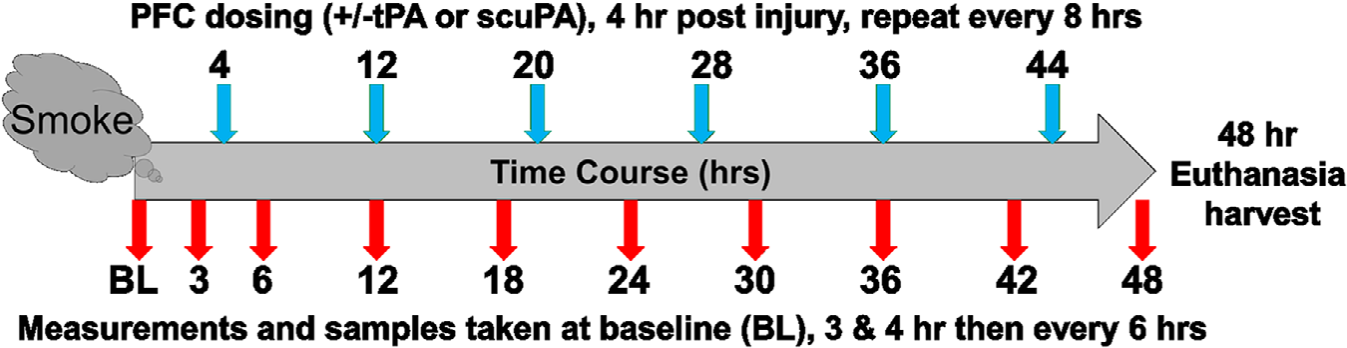
Schematic illustration of the experimental schedule. The gray line defines the 48‐h duration of the in vivo protocol, the gray cloud defines the smoke inhalation injury onset, red and blue arrows define measurement/sample and treatment intervals, respectively, following injury

All animal studies were approved by the Institutional Animal Care and Use Committees of The University of Texas Medical Branch at Galveston and The University of Texas Health Science Center at Tyler. Animals were managed according to the Guiding Principles in the Care and Use of Animals of the National Institutes of Health. Spontaneously breathing anesthetized adult female merino sheep were instrumented and exposed to cotton smoke‐induced ISALI (n = 36; 30‐45 kg; 6/group), as previously described.^7^, ^26^ Briefly, the sheep were surgically prepared for vascular access, pressure monitoring, blood sampling, and thermodilution cardiac output measurements under anesthesia and analgesia. They recovered for 5‐7 days. Then, on the day of the injury, pre and postinjury analgesia was provided with long‐acting buprenorphine, and the sheep were reanesthetized with isoflurane for placement of a tracheostomy tube, after which they were randomized by group. The experimental schedule is shown in Figure 1. The animals were supported with time‐cycled (20 br/min), volume‐controlled (12 mL/kg), pressure limited (35/5 cmH_2_O) mechanical ventilation (Hamilton G5 ventilator; Hamilton Medical, Inc., Reno, NV) with heated (33°C)/humidified inspired gas for a total of 48 h while conscious, as we previously reported.^7^, ^26^

Cardiopulmonary measurements (systemic and pulmonary arterial and capillary wedge blood pressures, heart rate, cardiac output, arterial and venous blood chemistry profile, airway pressures, and pulmonary mechanics) were recorded at baseline prior to injury (FiO_2_ = 0.21). While the sheep were anesthetized with isoflurane, smoke inhalation injury was induced using 48 breaths of cotton smoke (12 breaths × 4 sets) at < 40°C delivered with a modified bee smoker directly into the tracheostomy tube. Then, the animals were awakened and supported with FiO_2_ = 1 for the first 3 h after injury. Thereafter, FiO_2_ was adjusted to target oxygen saturation > 92% and PaO_2_ ∼ 100 mmHg, and respiratory rate was adjusted to target PaCO_2_ = 35‐45 mmHg. At 4 h post injury, the first dose of PFC (perfluoro‐octylbromide; FluoroMed L.P., Round Rock, TX) alone (8 mL) or PFC suspension of tPA (4 or 8 mg in 8 mL) or scuPA (4 or 8 mg in 8 mL) was delivered intratracheally at the ventilator wire during inspiration by syringe infusion. Dosing was repeated every 8 h, for a total of six 0.13 mL/kg doses. The dose of PA and volume of PFC were extrapolated from our previous study of aerosolization of the same PAs.^7^ Airway toilet (gentle bagging and in‐line suctioning) was performed as needed in response to alterations in respiratory patterns (eg, cough or sudden increases in airway pressure). The incidence of these events along with the presence of cast and exudate was recorded.

Cardiopulmonary measurements were repeated at least every 6 h for the duration of the 48‐h study. Animals were provided intravenous maintenance fluids (lactated ringer's 4 mL/h/kg with adjustments to keep hematocrit +/– 3%) and allowed free access to food but not water throughout the study period. Animals not treated with PFC served as controls for the effect of PFC alone.

The PaO_2_/FiO_2_ ratio, oxygenation index (OI), minute ventilation, and ventilatory efficiency index (VEI) were calculated to assess pulmonary gas exchange and ventilation. Briefly, OI and VEI reflect the gas exchange output relative to the ventilator input where OI = [(mean airway pressure × FiO_2_)/PaO_2_] × 100 and VEI = 3800/(peak inspiratory – peak expiratory pressure) × (breathing frequency) × PaCO_2_.^28^, ^29^ Actual tidal volume, breathing frequency, and calculated minute ventilation were evaluated as percentage of ventilator settings (“effective”). Hemodynamics were evaluated from measurements of systemic arterial, central venous, and pulmonary blood pressures; cardiac output; and calculations of systemic and pulmonary vascular resistance, respectively.

### 2.2 Preparation of PA therapies

A bulk solution of scuPA (NHLBI SMART) with specific enzymatic activity of ∼150 000 IU/mg, 99.5% purity 0.23 EU/mg bacterial endotoxin level,^7^ was used in Dulbecco's phosphate buffer to a concentration of 0.5 mg/mL of scuPA, which was then mixed with 1.5% w/v of mannitol. As such, mannitol was used as a stabilizing agent and carrier to prepare the PA lyophilized powder, prepared by our Pharmacology coauthors at the College of Pharmacy (The University of Texas at Austin, Austin, TX). It is often used in this manner and is used in inhalation medications without safety issues.

The solution was filtered through a 0.22 μm surfactant‐free cellulose acetate sterile syringe filter (Corning Inc., Corning, NY). Aliquots of 2 mL of the scuPA solution were filled into 5 mL borosilicate glass vials and lyophilized (VirTis Advantage Lyophilizer; VirTis Company, Inc., Gardiner, NY). The lyophilization cycle is shown in Table S1 (https://figshare.com/s/248b4e874f9d9dafb27e). PA/PFC suspensions were then prepared. tPA was single‐chain tPA; Cathflo Activase (Genentech, San Francisco, CA). Lyophilized tPA or scuPA was removed from vials and passed through a 30‐mesh sieve (particles < 0.6 mm). The tPA and scuPA powder were dispersed in PFC liquid at two concentrations, 0.5 and 1 mg/mL. Eight mLs of PA/PFC suspension (4 or 8 mg PA doses) were aliquoted and transferred into a 10‐mL borosilicate glass syringe. The syringes were sealed and stored at 2‐8°C until administration. Before administration, the syringes were warmed and then gently inverted back and forth for 1 min to ensure that the powder was redispersed.

### 2.3 Stability of PA activity in PFC preparations

Lyophilized powder of scuPA and Cathflo Activase before dispersion in the PFC was analyzed for the specific enzyme activity. The scuPA and tPA powder were dispersed in PFC at 0.5 mg/mL. The dispersions were collected and analyzed for specific enzyme activity after storage at 6‐10°C for 2 days and 25°C for 3 h. The dispersions were extracted by adding 100 µL of water into 500 µL of the dispersion. The dispersions were put on ice to separate the dispersion into two phases, including PFC phase (bottom layer) and aqueous phase (upper layer). The upper layer was collected and analyzed for the protein content by BCA assay and the enzyme activity by kinetic activity assay and then the specific enzyme activity was calculated as described previously.^30^

The specific enzyme activities of the PA/PFC formulation after dispersion and storage are shown in Table S2 (https://figshare.com/s/c77b522ff448341f596c). No significant changes were found over 48 h during storage, so that preparations were stable between preparation and use.

### 2.4 Histological analysis

Following euthanasia, the right lung was removed by thoracotomy. Airways were inspected for the presence of cast material. A 1‐cm‐thick section was taken from the middle of the right lower lobe, and the section was then immersion fixed with 10% formalin. Four samples (1 × 1 × 0.5 cm) of this fixed section were obtained using a predetermined matrix. These samples were processed, embedded in paraffin, and step‐sectioned at 500 µm intervals. Five 5‐μm sections from each sample were slide mounted and stained with hematoxylin and eosin. Analyses were performed while blinded to treatment group. Lung sections were first viewed through a transparent grid matrix at low power, photographed and digitized, and then changed to higher power to randomize selection. To eliminate sampling bias, every tenth grid region was then photographed and digitized, for a total of five higher power images/section.

Morphometric image analysis (Image Pro Plus, Silver Spring, MD) was performed with customized algorithms that were free of geometric assumptions to assess the tissue expansion index (ratio of volume of gas exchange to parenchymal space) by densitometry, to count the number of open gas exchange units per fixed field size using a modification of the radial alveolar count method,^18^, ^31^, ^32^, ^33^ and to assess quality and homogeneity of gas exchange unit expansion by the weighted mean of airspace diameters (D2).^34^, ^35^, ^36^, ^37^ In addition, all bronchi, bronchioles, and terminal bronchioles in these sections were identified, where bronchi were defined as airways with supporting cartilage and mucous glands, bronchioles as lacking cartilage and mucous glands, and terminal bronchioles as having short cuboidal lining cells and minimal connective tissue underlying the epithelium.^26^, ^27^, ^38^ These airways were quantitatively scored for lumen obstruction using Fiji‐enhanced ImageJ manual tracing of the inner lumen wall, background correction, and minimum thresholding to calculate the percentage of surface area of lumen obstruction by cast material.^39^

### 2.5 Bronchoalveolar lavage protocol and analyses

Bronchoalveolar lavage (BAL) was performed by wedging a Foley urinary catheter within a left lower lobe segment, introducing 50 mL of PBS via the catheter followed by gentle suctioning. The BAL yield (5‐20 mL) was centrifuged (2500 *g*; 4°C; 10 min), after which cell‐free lavage was immediately frozen and stored at −80°C. Measurements of the scuPA and tPA activity and antigen in BAL were done using ELISAs (Molecular Innovations, Novi, MI) per the manufacturer's protocol. Fibrinolytic activity in BAL was analyzed as we previously reported.^40^ Briefly, BAL fluids (0.1 mL) were added over a FITC‐fibrin film, which was preformed in the 96‐well plate. Fibrinolytic activity was monitored by changes in fluorescence emission at 510 nm (excitation 490 nm) with time. Plasminogen activation assays were performed as previously described.^40^ Levels of active PAI‐1 in the BAL samples were determined using an active rat PAI‐1 ELISA (Molecular Innovations) following the manufacturer's protocol.

### 2.6 Statistical analysis

Data and statistical analyses were performed using Microsoft EXCEL Groups and Graph Pad PRISM 6 (Graphpad Software, La Jolla, CA). Dependent variables were considered to be continuous and tested for normal distribution. A fixed‐effects model was used throughout. Each physiological variable was analyzed based on an ANOVA multifactorial design (time and treatment) with repeated measures on time. Treatment group means for the physiological data were compared vertically using the Dunn‐Bonferroni procedure and horizontally using linear contrast. Histological and biochemical parameters were analyzed using ANOVA Kruskal‐Wallis with multiple pairwise comparisons among all groups using Dunn's post test. Data are presented as mean ± SE, and significance was accepted at the *P* < .05 level.

## 3 RESULTS

### 3.1 Effects of PFC alone on pathophysiological and morphometric readouts

We first compared the effects of PFC administration alone to no PFC treatment in sheep with ISALI. Administration of PFC at the dosages and schedule used in this study did not exert any significant effect on the broad range of physiological readouts we assessed (Fig. S1) (https://figshare.com/s/5ab0ef77542f265aa4e6). There were likewise no effects of the delivery of PFC alone via the airways on the morphometric indices of histologic changes in the airways or lung parenchyma (Fig. S2) (https://figshare.com/s/0b9f4228f26da37e5f6e). To responsibly conserve animals and cost, we subsequently used PFC‐treated animals as vehicle controls for the interventions using PFC‐PA activator suspensions.

### 3.2 Overall management and qualitative assessment

The overall incidence of airway toilet for cough or transient elevation in airway pressures, or both, was 43% across all animals in the study, typically occurring 12 h or later after injury. There were group differences in this finding with none of the animals treated with PFC alone requiring intervention, while 50% of animals treated with tPA/PFC suspension and 40% of animals treated with scuPA/PFC suspension were treated. Cast retrieval before harvest occurred in 50% of PFC‐treated animals, 83% of those treated with either tPA/PFC or scuPA/PFC 4 mg suspension or tPA/PFC 8 mg suspension, and 33% when treated with scuPA/PFC 8 mg suspension. At postmortem dissection, airway casts beyond the mainstem bronchi were found in all of the animals treated with PFC only and tPA/PFC or scuPA/PFC suspension at 4 mg dose and in 50% of the animals treated with the PA/PFC suspensions at 8 mg.

### 3.3 Overall physiological phenotype of ISALI

**FIGURE 2:**
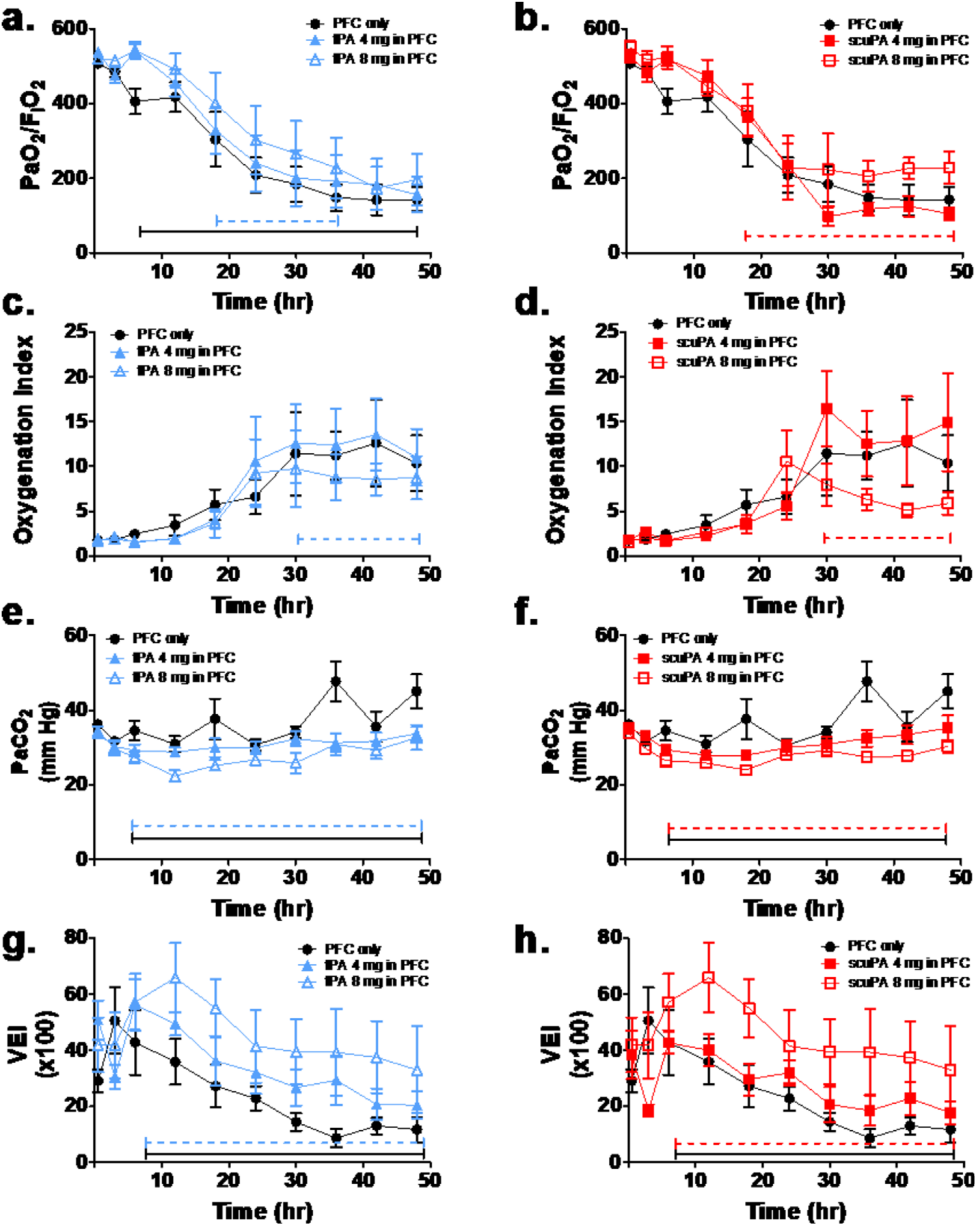
Dose‐response effects of perfluorochemical (PFC) tPA (blue: n = 6) and scuPA (red: n = 6) suspensions on indices of gas exchange; response to treatment to PFC only (black: n = 6) is shown as comparison. Dashed brackets demonstrate significant differences as a function of plasminogen activator (PA) dose (4 mg: solid symbols; 8 mg: open symbols); solid brackets demonstrate significant differences between PFC PA suspensions and PFC only. Data are shown as mean ± SEM. In comparison with baseline, following injury PaO_2_/FiO_2_ ratio (A and B) and ventilation efficiency index (VEI) ( G and H) decreased significantly and oxygenation index (C and D) increased significantly (*P* < .0001) over time independent of group. With treatment, there was a significant dose‐dependent improvement in oxygenation (greater PaO_2_/FiO_2_ ratio: *P* < .05; lower oxygenation index: C; *P* < .05; D; *P* < .01) and ventilation (lower PaCO_2_: *P* < .01; E and F; greater VEI: (G; *P* < .05; H; *P* < .01) for both PFC tPA and PFC scuPA suspensions most notably across parameters for scuPA 8 mg as compared with 4 mg and PFC only

**FIGURE 3:**
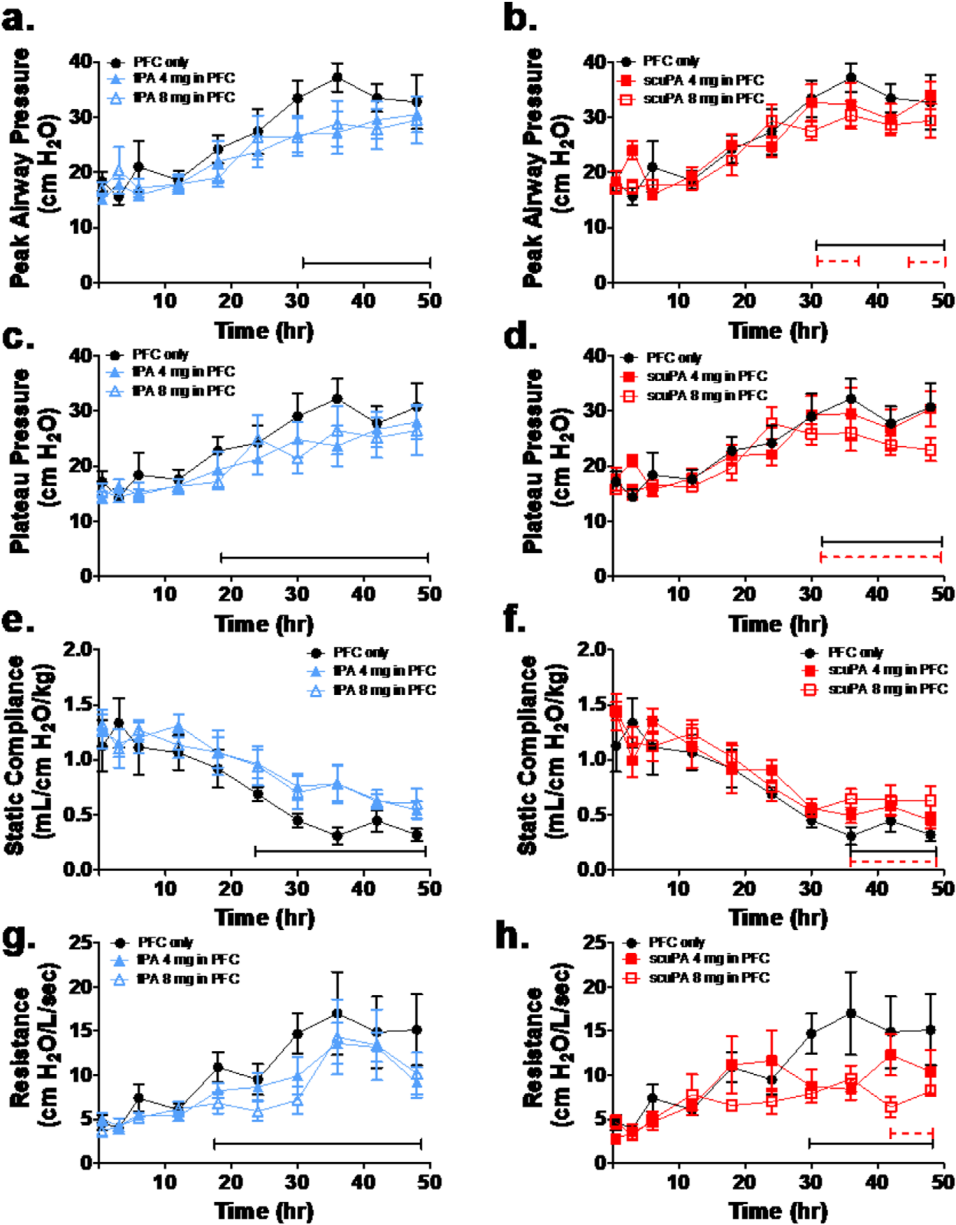
Dose‐response effects of perfluorochemical (PFC) tPA (blue: n = 6) and scuPA (red: n = 6) suspensions on indices of ventilatory support and pulmonary mechanics. The response to treatment to PFC only (black: n = 6) is shown for comparison. Dashed brackets demonstrate significant differences as a function of plasminogen activator (PA) dose (4 mg: solid symbols; 8 mg: open symbols); solid brackets demonstrate significant differences between PA/PFC suspensions and PFC only. Data are shown as mean ± SEM. In comparison with baseline, following injury peak, airway pressure (A and B), plateau pressure ventilation (C and D), and pulmonary resistance ( G and H) increased and compliance decreased (E and F) significantly (*P* < .0001) over time independent of group. With tPA/PFC suspension treatment, ventilatory pressures (A and C; *P* < .05) were lower and compliance (E; *P* < .01) was higher compared with PFC only over time, independent of tPA dose. With scuPA/PFC suspension treatment, there were significant dose‐dependent differences with lower ventilatory pressures (B and D; *P* < .05) and resistance (H; *P* < .01) and higher compliance (*P* < .05) for scuPA 8 mg as compared with scuPA 4 mg and PFC alone

**FIGURE 4:**
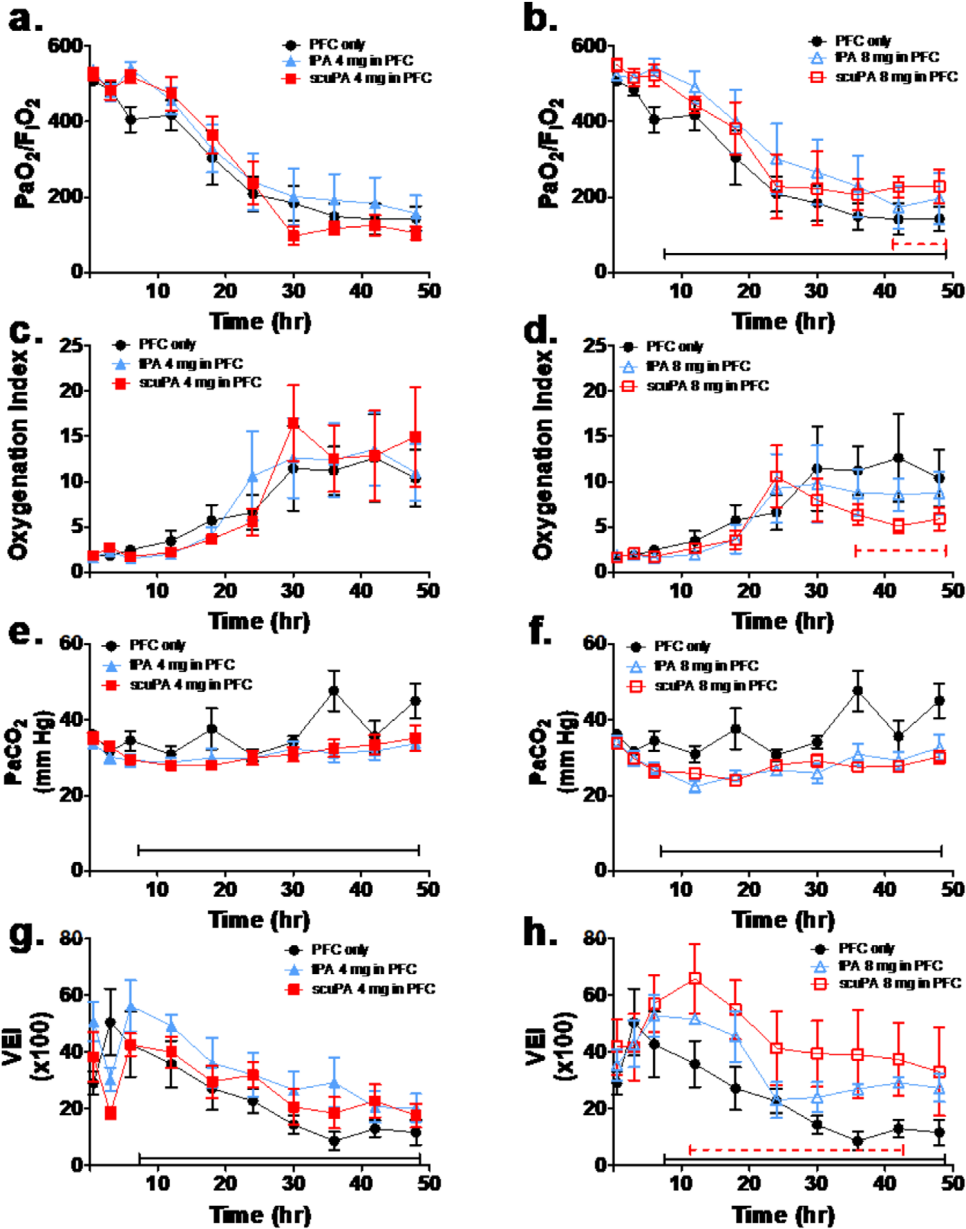
Plasminogen activator‐type effects, tPA (blue: n = 6) and scuPA (red: n = 6) PFC suspensions on indices of gas exchange. The response to treatment to PFC only (black: n = 6) is shown for comparison. Dashed brackets demonstrate significant differences as a function of type of plasminogen activator (PA); solid brackets demonstrate significant differences between PA/PFC suspensions and PFC only. Data are shown as mean ± SEM. With treatment, there was a significant PA type‐dependent improvement in oxygenation (B; greater PaO_2_/FiO_2_ ratio: *P* < .05; D; lower oxygenation index: *P* < .01) and ventilation (H; greater VEI: *P* < .01) that was also dose dependent favoring scuPA 8 mg/PFC suspension over tPA/PFC suspension and PFC alone

**FIGURE 5:**
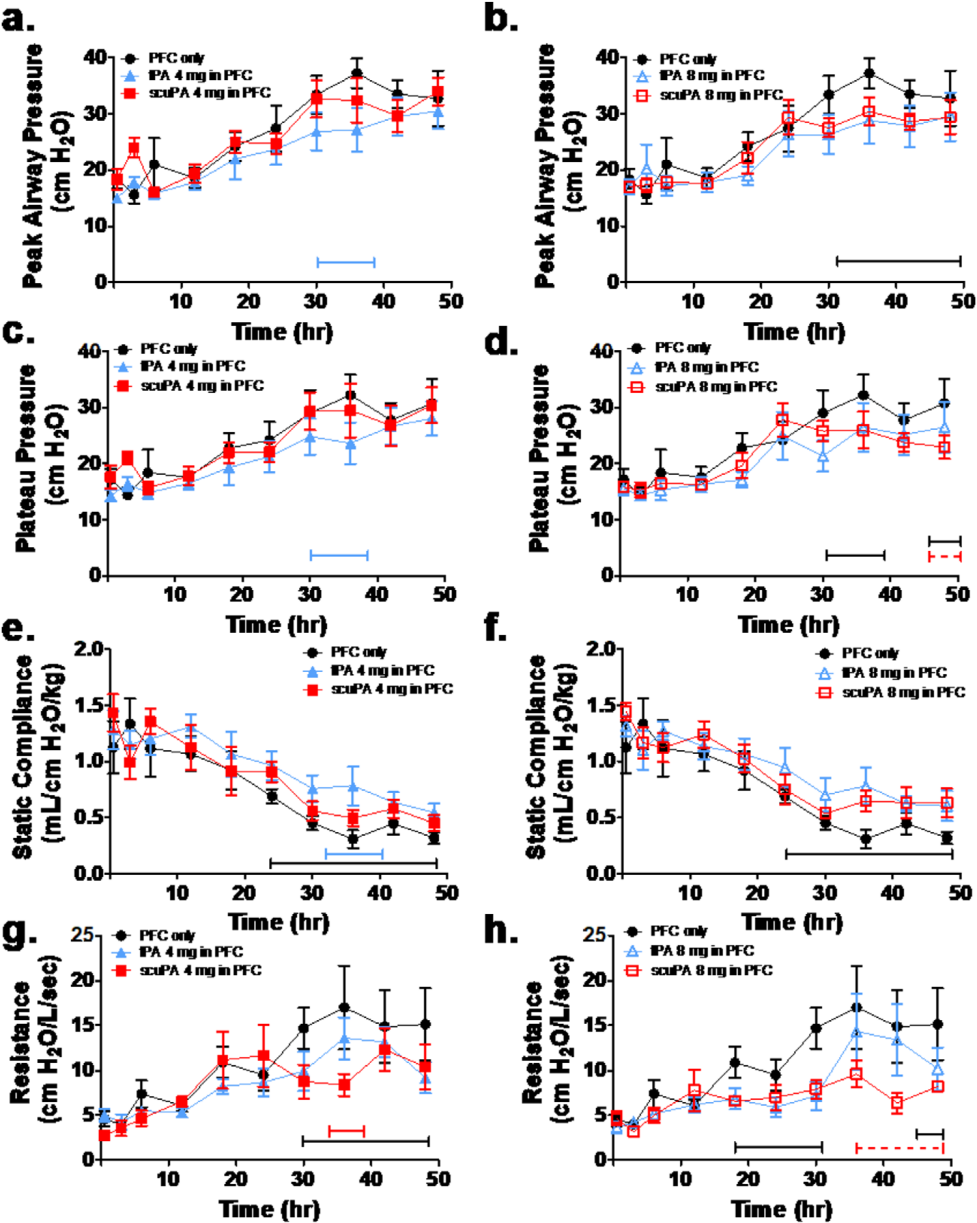
Plasminogen activator‐type effects, tPA (blue: n = 6) and scuPA (red: n = 6) PFC suspensions on indices of ventilatory support and pulmonary mechanics. The response to treatment to PFC only (black: n = 6) is shown for comparison. Dashed brackets demonstrate significant differences as a function of type of plasminogen activator (PA); solid brackets demonstrate significant differences between PA/PFC suspensions and PFC only. Data are shown as mean ± SEM. Ventilatory pressure support and pulmonary mechanics parameters demonstrated significant differences between the type of PA, dependent upon parameter and dose. At 4 mg, the PA type/PFC suspension‐dependent differences in peak airway (A; *P* < .05) and plateau pressure (C; *P* < .05), resistance (G; *P* < .01), and compliance (E; *P* < .05) were not sustained out to 48 h. In contrast at 8 mg, there was sustained significant reduction in ventilatory pressures (B and D; *P* < .05) and resistance (H; *P* < .01) decreasing more (*P* < .05) with scuPA/PFC suspension as compared with tPA/PFC suspension and PFC alone and improvement in compliance (F; *P* < .05) compared with PFC alone

The physiological profiles are shown in Figures 2 through 5 and Tables S3 (https://figshare.com/s/f84bcadd9174078d8ac4) and S4 (https://figshare.com/s/2c9007ea937f8dd962e1). There were no significant group differences in gas exchange (Figures 2 and 4), ventilatory support, and pulmonary mechanics (Figures 3 and 5, Table S3) and hemodynamics (Table S4) at baseline or after injury, pretreatment (3‐h time point). All gas exchange (Figures 2 and 4), ventilatory support, and pulmonary mechanics parameters (Figures 3 and 5, Table S3) changed significantly after injury compared with baseline and as a function of time after injury, independent of group. Specifically, oxygenation was impaired as indicated by decreased PaO_2_/FiO_2_ ratio and increased OI. Ventilation was also impaired reflected by increased PaCO_2_ and decreased VEI. The injury was also associated with increased ventilator support requirements (peak and plateau airway pressures) and impairment of pulmonary mechanics, including decreased compliance and increased resistance over time. Hemodynamics parameters (Table S4) demonstrated significant differences after injury compared with baseline and as a function of time after injury, independent of group. Cardiac output increased significantly after injury, pretreatment, as compared with baseline, and then decreased back toward baseline over time in all PA/PFC suspension‐treated groups. Similarly, there was a significant but small increase in systemic arterial pressure and central venous pressure post injury before treatment compared with baseline. These changes reverted toward baseline over time in all PA/PFC suspension‐treated groups. There were no significant differences in systemic vascular resistance between baseline and injury before treatment or over time following treatment, independent of group. Relative to central and systemic hemodynamics, pulmonary arterial and pulmonary capillary wedge pressures showed significant and greater increments post injury before treatment versus baseline. Following PA/PFC suspension treatment, these changes over baseline were sustained over time. Pulmonary vascular resistance increased over time, independent of group.

### 3.4 Dose‐response effects of tPA/PFC and scuPA/PFC suspensions on gas exchange, ventilatory support, and pulmonary mechanics

The dose‐response effect of tPA/PFC and scuPA/PFC suspensions on gas exchange is shown in Figure 2. Effects of the PA/PFC suspensions on ventilatory support and pulmonary mechanics are shown in Figure 3 and Table S3. Gas exchange parameters demonstrated a significant dose‐dependent improvement in oxygenation (Figures 2A and B: greater PaO_2_/FiO_2_ ratio; Figures 2C and D: lower oxygenation index) and ventilation (Figures 2E and F: lower PaCO_2_; Figures 2G and H: greater VEI, independent of the type of PA), in favor of scuPA 8 mg/PFC suspension as compared with scuPA 4 mg/PFC suspension and PFC only. Similar but temporally more limited changes were observed in favor of tPA 8 mg/PFC suspension. Ventilatory pressure support (Figures 3A and C), tidal volume, breathing frequency, and minute ventilation (Table S3) and pulmonary mechanics (Figures 3E and G) parameters demonstrated significant differences between tPA/PFC suspension treatments and PFC only. Ventilator pressures and “effective breathing frequency and minute ventilation” were lower, and compliance and “effective tidal volume” were higher in tPA/PFC suspension treatment groups as compared with PFC only over time, independent of tPA dose. In contrast, with scuPA/PFC suspension, significant dose‐dependent differences were seen with lower ventilatory pressures (Figures 3B and D) and resistance (Figure 3H), and higher compliance (Figure 3F) was observed when scuPA 8 mg/PFC suspension was used as compared with scuPA 4 mg/PFC suspension and PFC only. Significantly higher “effective tidal volume and minute ventilation” (Table S3) was observed in sheep treated with scuPA 8 mg/PFC suspension as compared with scuPA 4 mg/PFC suspension.

Overall, the gas exchange, ventilatory support, and pulmonary mechanics data demonstrated a superior aggregate dose response in favor of scuPA 8 mg/PFC suspension over lower dose scuPA/PFC suspension, either tPA dose/PFC suspension or PFC only.

### 3.5 Dose‐response effects of tPA/PFC and scuPA/PFC suspensions on hemodynamics

The dose‐response effect of tPA/PFC and scuPA/PFC suspensions on hemodynamics is shown in Table S4. Post injury after initiation of PA/PFC suspension treatment, cardiac output and central venous pressure were lower than in the PFC only group, independent of time and tPA/PFC or scuPA/PFC suspension dose. While returning toward baseline values, there were no significant dose‐dependent differences in systemic arterial pressure and systemic vascular resistance for either tPA/PFC or scuPA/PFC suspension‐treated animals or how each PA/PFC suspension dose response compared with PFC‐only‐treated animals. In this regard, postinjury systemic vascular resistance by 12‐18 h was greater in PA/PFC suspension‐treated animals as compared with those treated with PFC only, independent of the PA suspension dose. Similarly, after PA/PFC suspension treatment, there were no significant dose‐dependent differences in the sustained injury‐induced increase in pulmonary arterial and pulmonary capillary wedge pressure; the further increase in pulmonary vascular resistance over time was mitigated with scuPA 8 mg/PFC suspension relative to scuPA 4 mg/PFC suspension.

### 3.6 PA/PFC suspension type (tPA vs scuPA) effects on gas exchange, ventilatory support, and pulmonary mechanics

The effect of the type of PA/PFC suspension on gas exchange is shown in Figure 4 and on ventilatory support and pulmonary mechanics in Figure 5 and Table S3. All parameters changed significantly as a function of time after injury, independent of group. Gas exchange parameters demonstrated significant PA type/PFC suspension‐dependent improvements in oxygenation (Figures 4A and B: greater PaO_2_/FiO_2_ ratio and Figures 4C and D: lower oxygenation index) and ventilation (Figures 4E and F: lower PaCO_2_ and Figures 4G and H: greater VEI) that were dose dependent. At 8 mg, oxygenation and VEI (Figures 4D and H) were superior with scuPA/PFC compared with tPA/PFC suspension or PFC only. As shown in Figure 5 and Table S3, ventilatory pressure support and pulmonary mechanics parameters demonstrated significant differences between the type of PA/PFC suspension, dependent upon parameter and dose. At 4 mg, PA type/PFC suspension‐dependent differences in ventilatory pressures (Figures 5A and C), resistance (Figure 5G), and compliance (Figure 5E) were not maintained to 48 h. By contrast at 8 mg, there were sustained reductions in ventilatory pressures (Figures 5B and C) and resistance (Figure 5H) decreasing significantly more with scuPA/PFC than tPA/PFC suspension or PFC alone. Improvement in compliance (Figure 5F) and “effective tidal volume and minute ventilation” (Table S3) was superior with scuPA/PFC suspension as compared with tPA/PFC suspension.

Overall, the gas exchange, ventilatory, and pulmonary mechanics data again demonstrated a superior response in favor of scuPA/PFC suspension over tPA/PFC suspension with more sustained differences at the 8 mg dose.

### 3.7 PA/PFC suspension type (tPA vs scuPA) effects on hemodynamics

The effect of the type of PA/PFC suspension on hemodynamics is shown in Table S4. Post injury after initiation of PA/PFC suspension treatment, cardiac output and central venous pressures were lower than in the PFC‐only group, independent of time and type of PA/PFC suspension, and not significantly different between types of PAs. There were no significant PA type/PFC suspension‐dependent differences in systemic arterial pressure and systemic vascular resistance. Systemic vascular resistance was significantly greater in all PA/PFC suspension‐treated animals compared with PFC‐only‐treated animals.

In contrast to central and systemic hemodynamics, there was a significant PA type/PFC suspension‐dependent difference in the sustained injury induced increase in pulmonary arterial and pulmonary capillary wedge pressure and further increase in pulmonary vascular resistance over time. Specifically, these postinjury, posttreatment responses were more attenuated by scuPA/PFC relative to tPA/PFC suspension treatment.

### 3.8 Histology and morphometrics

**FIGURE 6:**
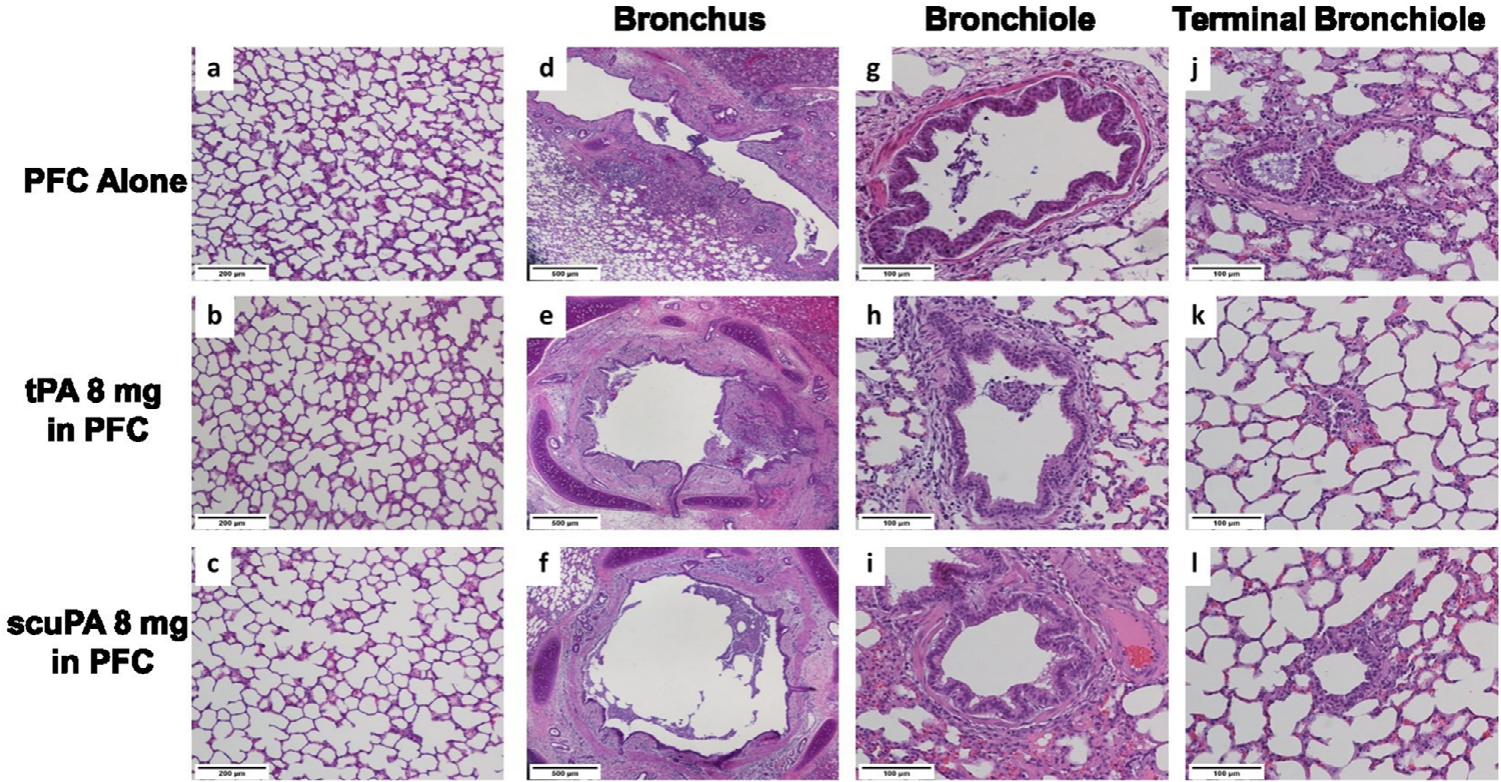
Representative light micrographs of hematoxylin and eosin lung and regional airway tissues. Images from lung parenchyma (A‐C) show characteristic changes of various degrees of nonhomogenous parenchymal hemorrhage, septal infiltrates, and hyaline membranes, characteristic of the ISALI model. Regional differences in cast debris are noted within each study group (bronchus: D‐F; bronchiole: G‐I; and terminal bronchiole: J‐L) with qualitative assessment of greater amounts in the bronchiole level. Scale bars reflect different levels of magnification between the lung tissue and regional airways

**FIGURE 7:**
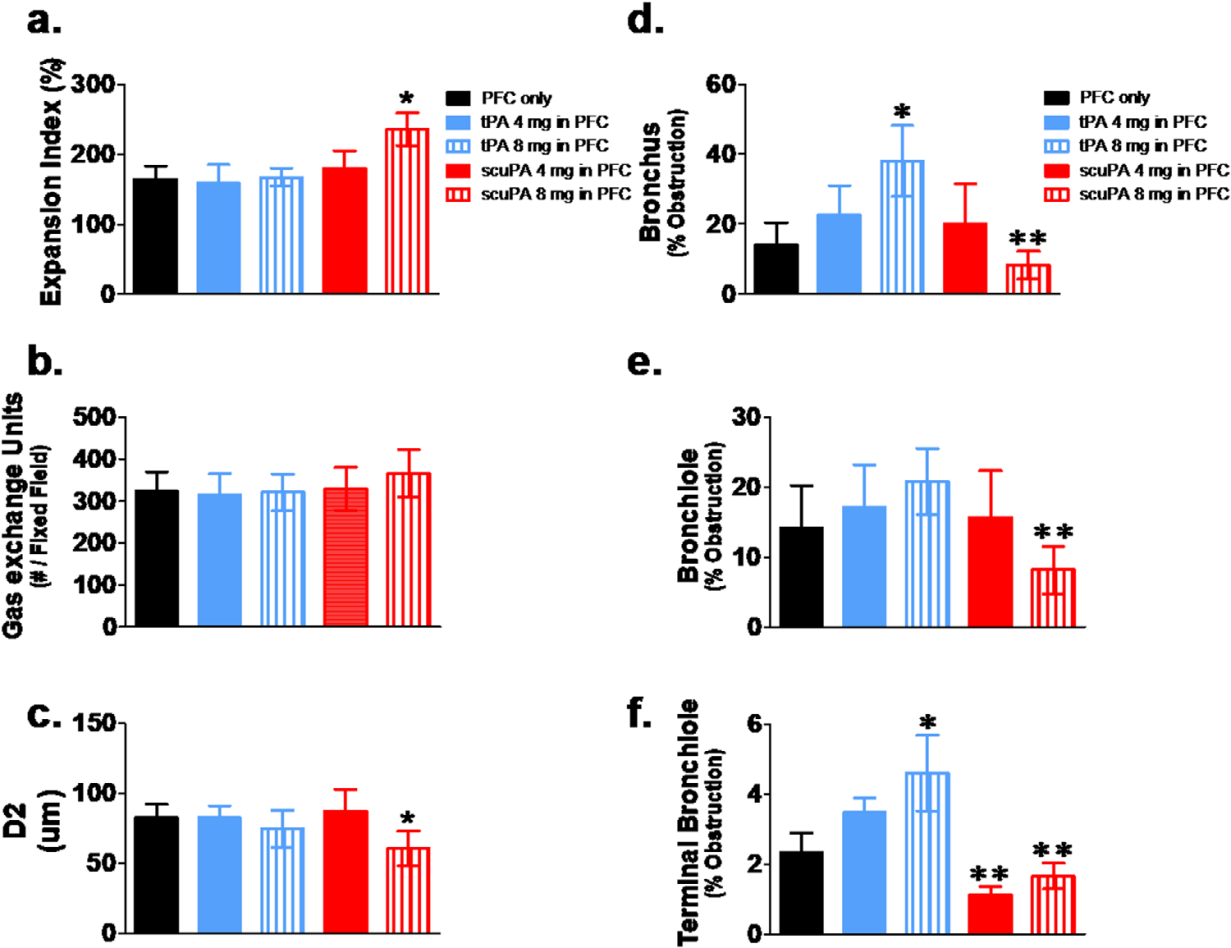
Quantitative histomorphological profile. A, Expansion index; B, number of opened gas exchange units per fixed field; C, D2, representing homogeneity of gas exchange unit expansion^34^, ^35^, ^36^, ^37^; D, bronchus % obstruction; E, bronchiole % obstruction; and F, terminal bronchiole % obstruction. Data are shown as mean ± SEM. Treatment with PFC only: black bars; tPA 4 mg/PFC suspension: blue bars; tPA 8 mg/PFC suspension: blue vertical striped bars; scuPA 4 mg/PFC suspension: red solid bars; scuPA 8 mg/PFC suspension: red vertical striped bars. Data demonstrate significantly greater (*P < .05) lung tissue expansion and homogeneity (lower D2) of expanded gas exchange units following treatment with scuPA 8 mg/PFC suspension as compared with all other groups. Large airway (bronchus: D) and small airway (bronchiole: E; terminal bronchiole: F) obstructions were significantly (*P < .05) greater in animals treated with tPA 8 mg/PFC suspension as compared with all other groups. Bronchus and bronchiole obstruction was lowest (**P < .01) following scuPA 8 mg/PFC suspension treatment than all other groups with scuPA versus tPA at either dose. Terminal bronchiolar obstruction was significantly lower (**P<.01) with scuPA/PFC suspension as compared with tPA/PFC suspension, independent of dose

Figure 6 illustrates the representative light micrographs of hematoxylin‐ and eosin‐stained lung sections and airway generations from animals 48 h post injury, which received equivalent dosing of tPA/PFC suspension or scuPA/PFC suspension or PFC alone in equivalent low volumes of PFC. Tissues from all animals had characteristic changes associated with ISALI with various degrees of nonhomogenous parenchymal lung hemorrhage, septal infiltrates, and hyaline membranes. There was no evidence of significant airway hemorrhage at any airway level in any of the groups, consistent with absence of airway bleeding in vivo. Regional differences in the amount of airway casts were qualitatively greater at the bronchiolar level. Quantitative data of parenchymal and airway images from animals in all groups are shown in Figure 7. These analyses demonstrated significantly greater lung tissue expansion (Figure 7A: expansion index) and homogeneity (Figure 7B: lower D2) of expanded gas exchange units in sheep treated with scuPA/PFC suspension. There was a trend toward a greater number of expanded gas exchange units following treatment with scuPA 8 mg/PFC suspension compared with all other groups.

The number of airways evaluated in the tissue sections was significantly different as a function of generation (*P* < .01; bronchi 13 ± 4 SEM, bronchioles 223 ± 28 SEM, terminal bronchioles 51 ± 7 SEM) but not as a function of group. As shown in Figure 7, there were regional differences in airway obstruction; % lumen obstruction decreased from proximal to distal airways, independent of group. Treatment with tPA 8 mg/PFC suspension resulted in greater large (Figure 7D: bronchus) and small airway (Figure 7E: bronchiole; Figure 7F: terminal bronchiole) obstruction than in all other groups. Terminal bronchiole airway obstruction was lower with scuPA/PFC suspension versus tPA/PFC suspension at either dose. Overall, both large and small airway obstructions were significantly lower with scuPA 8 mg/PFC suspension compared with tPA/PFC suspension treatment, independent of tPA/PFC suspension dose, and consistent with a reduction in cast burden.

### 3.9 Biochemical analyses

**FIGURE 8:**
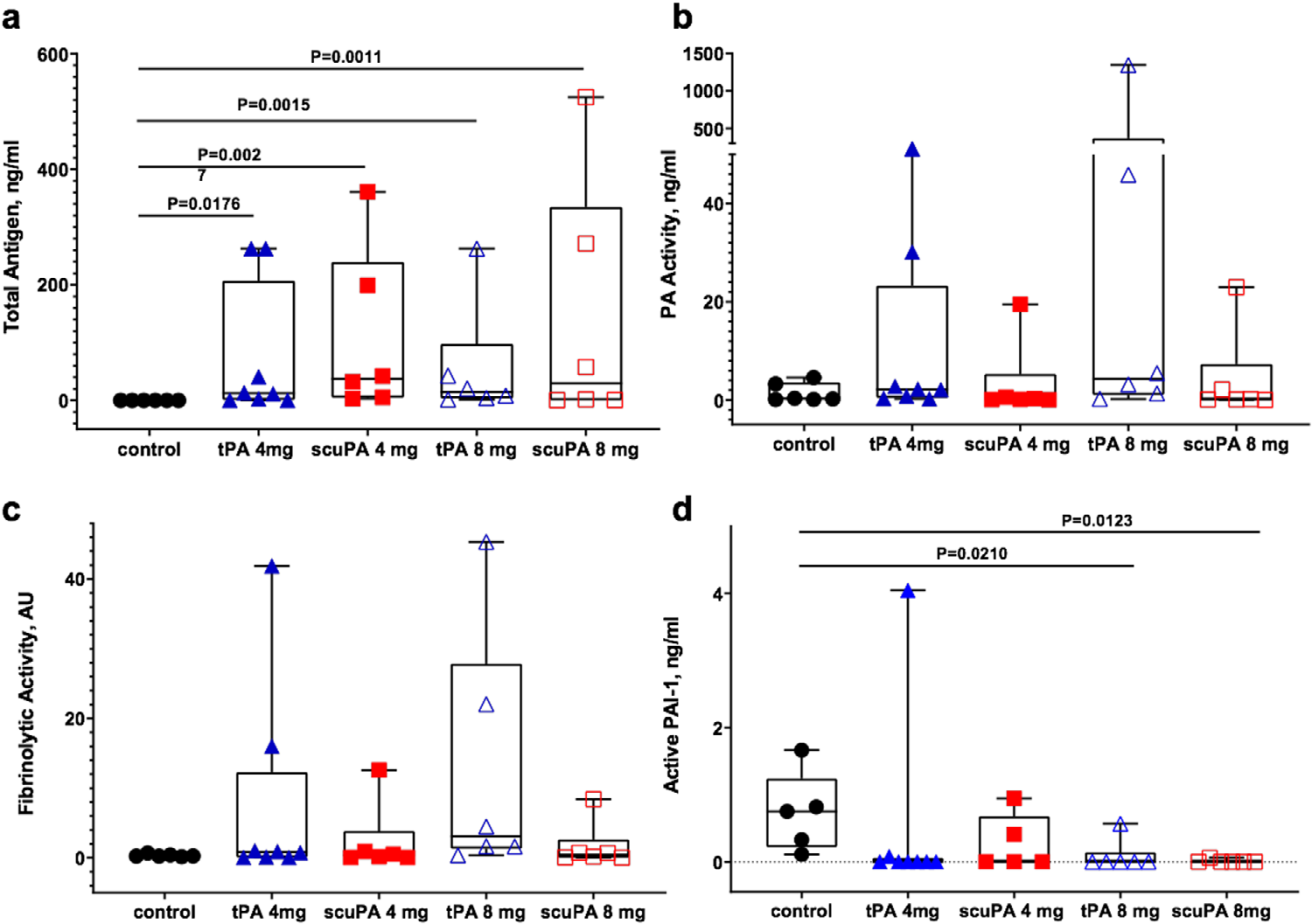
Biochemical analyses of bronchoalveolar lavage fluids (BALFs). Human tissue and urokinase plasminogen activators are detected in BAL of the sheep treated with perfluorochemical (PFC) tPA (blue: 4 mg treatment n = 8; 8 mg treatment n = 6), scuPA (red: n = 6) suspensions. BAL of sheep treated with PFC/PA (red and blue) and PFC alone (black: n = 6) was assayed for the presence of human tPA or scuPA antigens (A), plasminogen activating (B) and fibrinolytic (C) activities, and level of active PAI‐1 (D). While human scuPA or tPA was not detected in animals treated with PFC alone (black circles), an increase in doses of tPA (blue) or scuPA (red) (4 mg: solid symbols; 8 mg: open symbols) resulted in the apparent increase of BALF human plasminogen activator antigens. Plasminogen activating and fibrinolytic activities were variably increased in BALF of animals treated with tPA/PFC suspension (B and C). Level of active PAI‐1 (D) in BALF of PFC or PA/PFC suspension‐treated animals was analyzed using rat PAI‐1 ELISA (Molecular Innovations). PAI‐1 activity was at or below the level of detection in BALF of sheep treated with 8 mg of either scuPA or tPA/PFC suspensions. One data point in the control group (black circles) was omitted. Data are presented as box plots, showing median and interquartile ranges. Statistically significant differences are noted. There were no significant differences in levels of PA or fibrinolytic activities between the studied groups

Samples of BAL collected at 48 h (Figure 1), 4 h after the last (sixth treatment), were used to confirm the successful pulmonary delivery of PA/PFC suspensions. Samples of BAL were first analyzed for levels of tPA and uPA antigen (Figure 8A). The concentration of total antigen trended higher in BAL of the animals treated with scuPA 8 mg/PFC suspension than that of tPA 8 mg/PFC suspension (Figure 8A). PA activity (Figure 8B) in BAL measured independently by commercial ELISA (not shown) or plasminogen‐activating assay,^41^ or both, demonstrated variability with trends toward increased PA activities in BAL of animals treated with either PA (Figure 8B).

Levels of fibrinolytic activity in BAL samples (Figure 8C) demonstrated comparable trends and correlated with PA activity (Figure 8B). Notably, no significant PA or fibrinolytic activity was detected in BAL of the control animals treated with PFC alone. Supplementation of samples possessing no PA activity with 10 nM of exogenous tPA resulted in fibrinolytic activity reflecting activation of accumulated endogenous plasminogen (not shown). Active PAI‐1 was significantly higher in the group treated with PFC alone than the groups treated with either 8 mg of scuPA/PFC suspension or tPA/PFC suspension, where active PAI‐1 was at the limit of detection in the majority of the samples (Figure 8D). These data indicate that active PAI‐1 was largely quenched by the PA/PFCs and that overexpression of endogenous PAI‐1 contributes to the suppression of PA and fibrinolytic activity found in the majority of the samples (Figures 8B and C).

## 4 DISCUSSION

The central findings of this study indicate that in a translational sheep model of ISALI, low‐volume repeated PFC‐facilitated PA delivery improved physiologic and histologic outcomes. Administration of scuPA/PFC suspension at the 8 mg dose resulted in reduced cast burden and sustainable improvement in physiologic outcomes compared with low‐volume PFC alone. Further, dose‐response and PA‐type response analyses demonstrated the relative superiority of scuPA 8/PFC suspension mg versus lower dose scuPA/PFC suspension or tPA/PFC suspension at comparable doses in terms of physiologic and histological outcomes.

ISALI affects thousands of smoke‐exposed patients in civilian and military practice annually,^4^ contributing to greater than 3000 deaths and 17 000 fire‐related injuries in the United States annually and a fire‐related mortality rate of 2‐3/100 000 population.^42^ It is characterized by airway epithelial lining damage promoting leakage of plasma substrates into the large airways with coagulation suggesting that endogenous fibrinolytic activity is locally impaired. Aberrant fibrin turnover occurs in ISALI with fibrin casts originating in the large airways that are prone to distal migration with alveolar deposition.^4^, ^38^ These derangements compromise pulmonary mechanics by increasing resistance and decreasing compliance, leading to nonhomogenous lung expansion and distribution of ventilation, atelectasis, and ultimately impaired gas exchange.

Interventions to mitigate the development of airway casts or remove established cast formations are limited. Clinical efficacy of nebulized anticoagulants remains unproven, and they carry risks of initiating local or systemic coagulopathy.^43^, ^44^, ^45^ Supportive management is suboptimal. Clinical outcomes reflect significant morbidity, including bronchial reactivity and pulmonary fibrosis, and is not uncommonly associated with progression to acute respiratory distress syndrome (ARDS) with mortality rates approaching 40%.^46^

Anticoagulants do not support the clearance of established casts, but PAs degrade fibrin deposits by generating increments of plasmin. Because smoke‐induced local impairment of fibrinolytic activity contributes to deposition and persistence of these casts, we previously treated the sheep model of cotton ISALI with nebulized tPA or scuPA to assess the safety and physiologic efficacy to reduce cast burden.^7^ We found that nebulized tPA delivery was confounded by airway bleeding, while scuPA was well tolerated and supported transient improvement in gas exchange and lung mechanics. tPA is an active protease/fibrinolysin that avidly binds fibrin and is rapidly inactivated by PAI‐1 or other inhibitors in ALI. Nebulized tPA doses that overwhelm local inhibitors predispose to airway bleeding.^7^ scuPA, a more bioavailable agent, forms bioactive complexes with α‐macroglobulins, which are resistant to inhibition by PAI‐1. These complexes durably release low levels of active PA to mitigate bleeding risk.^47^ These differences may explain the observed PA type‐dependent differences in airway bleeding. The transient improvements in ISALI that could be achieved by nebulized scuPA or tPA may have been attributable to impaired distribution of the inhaled aerosolized PAs. We reasoned that a PFC delivery system would favor better airway and lung parenchymal distribution of exogenous PAs and improved outcomes in ISALI. We also inferred that PFC delivery would prevent excessive local accumulation of PAs to mitigate bleeding risk within the airways or lung parenchyma. We found that PFC delivery of the PAs was well tolerated and enabled durable outcomes that could not be achieved by nebulization of the agents at the same doses.^7^ Historically, intrapulmonary volumes of PFC liquids have been used as imaging agents as well as an assisted “liquid ventilation” approach in translational preclinical and clinical studies to improve gas exchange and alveolar recruitment at reduced ventilator pressures. Airway delivery of PFCs has also been used to remove airway and parenchymal debris in diverse forms of ALI and ARDS.^18^, ^21^, ^22^, ^23^, ^31^, ^48^, ^49^, ^50^, ^51^, ^52^ In this approach, various volumes of neat or aerosolized PFC are instilled and maintained at a reference lung volume until recovery to full gas breathing alone. The preponderance of studies confirms substantive benefits of PFCs in rescue of gas exchange, lung function, and mortality.^19^, ^21^, ^22^, ^53^, ^54^ PFCs have also been used to formulate drugs for delivery and distribution in the lung during “liquid ventilation,” including the recombinant protein superoxide dismutase, antibiotics, vasoactive substances, or surfactant in the formulation.^9^, ^11^, ^12^, ^13^, ^14^, ^15^, ^16^, ^55^ PFCs are also well tolerated and have direct beneficial effects in the compromised lung (decreased edema and inflammation) independent of the drug delivered.^18^, ^20^, ^51^ Given these advantages, we inferred that PFCs would improve airway delivery of PAs and improve outcomes in ISALI.

Unlike previous studies of PFC “liquid ventilation” or PFC‐facilitated pulmonary drug administration, the current experimental design utilized extremely low volumes of PFC as a distribution aide for the PAs. Our choice of the specific PFC (perfluoro‐octylbromide; perflubron) as a vehicle for intrapulmonary delivery is based on the supportive physicochemical properties and tolerance demonstrated in preclinical and clinical experiences when used for multiple different pulmonary applications (eg, imaging, ventilatory assist, and drug delivery).^10^, ^12^, ^22^, ^23^, ^48^, ^51^, ^52^ Relative to the low volumes of PFC alone, PAs delivered in the same volume of PFC demonstrated improvements in gas exchange, ventilatory support, pulmonary mechanics, and histologic outcomes. Dissolution and dislodgement of the airway casts appeared to contribute to these benefits.

Mechanistically, the respiratory gas solubility in PFC supports gas exchange during delivery. Unlike during “liquid ventilation,” where maintenance volumes of PFC effectively recruit lung volume, it is unlikely that the low volumes used in PA/PFC delivery were responsible for physiologic or cast‐burden improvements. This interpretation is supported by the lack of discernible effects in the injured non‐PFC‐treated animals versus those treated with low‐volume PFC alone. Rather, we reason that because of the low surface tension of the PFC liquid, the PFC distributed the PAs throughout the airways both on the luminal surface and between casts and airway wall, degrading casts as they formed while impeding formation of new casts. As the PFC volatized from the lung between dosing, the PAs remained to further act to dissolve airway casts and alveolar fibrin. Upon redosing with PA/PFC suspension, the low PFC volumes not only deposited additional drug but may have promoted cast removal by suctioning or cough. Because the PFC is incompressible, we infer that residual, nonvolatilized PFC promoted small airway patency, aiding alveolar recruitment. We speculate that reduced small airway obstruction, supporting improvements in resistance, compliance, oxygenation, and ventilation, likely contributed to the salutary effects of the PA/PFC suspension.

The bronchoalveolar lavage fluid (BALF) biochemical studies confirmed successful airway delivery of tPA and scuPA as PFC suspensions. The levels of total uPA antigen in both 4‐ and 8‐mg doses were comparable with those observed previously with nebulized PAs.^7^ Thus, both methods of delivery (nebulizing up to 44 mg of PA^7^ or delivery of 24 or 48 mg of PAs as a suspension in PFC; present study) resulted in similar levels of the antigen in the BALF. However, local delivery of PA by PFC delivery was considerably faster than by nebulization, likely mitigating PA inactivation by less efficient delivery of nebulized PA.^7^ The observed simultaneous increase in the PA and fibrinolytic activity in BALF (Figures 8B and C) indicates that PA/PFC suspension delivery can activate the endogenous fibrinolytic system and promote fibrinolysis of airway casts. The variability of detectable PAs and their activities in BALF likely reflects the anticipated differences in retrieval of the administered BAL or other effects that the PFC may have exerted. Because BALF was collected 4 h after the last administration of PA/PFC suspension, detection of the BALF antigens and PA or fibrinolytic activities likely became more difficult. A potential alternate effect leading to the variability of PA detection may have been greater retention of the administered BAL via increased small airway patency. On the other hand, there was accumulation of plasminogen in the BAL samples, which possessed no PA activity. This was confirmed by the activation of endogenous plasminogen with ex vivo supplementation of BALF by tPA, resulting in robust fibrinolytic activity in each BALF sample. This observation also suggests that PA activity may have been generated earlier within lower airway fluids and dissipated by the time of the BAL procedure. The results of the present study clearly indicate that (a) direct delivery of the PAs as a suspension in PFC is more effective than nebulization of the same amount of the drug, and (b) that a short half‐life of the PAs in vivo results in their fast inactivation and accumulation of endogenous plasminogen. Thus, delivery and retention of the PA activity at the site of the smoke inhalation injury are critical for successful therapy. Levels of PAI‐1 activity were reduced in PA/PFC suspension‐treated animals versus controls, suggesting that the inhibitor bounds the administered PAs, thereby reducing the detectable activity. Interestingly, higher levels of PA and fibrinolytic activity in BAL samples of animals treated with tPA/PFC suspension (Figures 8B and C) did not correlate with physiological outcomes, where scuPA 8 mg/PFC suspension demonstrated superiority over any other treatment. This could reflect differences in airway/alveolar processing of tPA and scuPA/PFC suspensions, which require further investigation beyond the scope of this study.

Overall, we found that both large and small airway obstructions were lower with the higher dose of scuPA/PFC suspension tested compared with tPA/PFC suspensions, independent of tPA dose and PFC only. These findings are consistent with (a) the parenchymal histological outcome of greater and more homogenous lung expansion and (b) associated functional outcome of lower resistance and greater compliance requiring less ventilatory pressure support to sustain improvements in oxygenation and ventilation. These reduced ventilatory requirements were also coupled to relative improvements in pulmonary hemodynamics. Notably, the treatment with scuPA/PFC suspension attenuated the injury‐induced increase in pulmonary arterial and capillary wedge pressure, as well as the increase in pulmonary vascular resistance over time. Compared with PFC alone in which arterial carbon dioxide tension continued to increase despite increasing ventilatory pressure support after injury, arterial carbon dioxide tension in the PA/PFC suspension‐treated animals was maintained with less ventilatory pressure support. There was also more “effective tidal volume and minute ventilation,” and greater ventilation efficiency, at the higher scuPA/PFC suspension dose. Collectively, these findings reflect the reduction in cast burden that occurred with the higher 8‐mg unit dose of scuPA/PFC suspension. Our finding that cast retrieval prior to and at harvest was lowest in animals treated with scuPA 8 mg/PFC suspension supports the concept that this intervention best mitigates cast formation and resolution/removal of casts. It is noteworthy to mention that the histologic data were derived from assessment of the findings in a single lobe, as described in the Methods. As such, these findings may not reflect the overall response of the lungs to the intervention as shown in the results. Further, the absence of airway bleeding in this study with either PA/PFC suspension may reflect improved distribution of PA by the PFCs as shown previously in the delivery of other biologics.^9^, ^10^ We speculate that improved distribution of the PAs as well as the effect of PA/PFC suspension “coating” the casts and airway contributed to the protective effects of the PA/PFC suspensions.

Finally, the dose‐response and PA‐type comparisons demonstrated the relative superiority with the highest dose (8 mg) of scuPA/PFC suspension tested in this study over lower doses. Furthermore, scuPA results exceeded tPA dose responses in gas exchange, ventilatory support, pulmonary mechanics, and histologic outcomes. It is noteworthy that there is a dose‐dependent sustained in vivo physiologic improvement and complementary superior histological outcome in the absence of airway bleeding with low‐volume PFC‐facilitated distribution of scuPA 8 mg/PFC suspension, as compared with tPA/PFC suspensions or previously studied aerosolized tPA or scuPA.^7^ In contrast to aerosolization or nebulization techniques in which a small percentage of the dose emitted from the device may be actually delivered to the lung, the entire dose is delivered to the lung using intratracheal PA/PFC delivery methods. It is conceivable that outcomes may be optimized further by expanded PA/PFC regimes, such as by increased concentration per dose or frequency of dosing.

## 5 CONCLUSIONS

The sustained salutary responses to scuPA/PFC suspension delivery are encouraging and support the possibility that the observed benefits could be of translational importance. This possibility would require future testing.

## CONFLICTS OF INTEREST

The views expressed in this article do not reflect the views of the authors’ institutions or the NHLBI and are solely those of the authors. Dr. Idell serves as an unpaid member of the Board of Directors, Founder and Chief Scientific Officer of Lung Therapeutics, Inc. (LTI) and has an equity position in the company, as does the University of Texas Horizon Fund and The University of Texas Health Science Center at Tyler. LTI is commercializing single‐chain urokinase for use in pleural injury and has licensed a patent for its use in the airway. He has conflicts of interest plans acknowledging and managing these declared conflicts of interest through The University of Texas Health Science Center at Tyler (UTHSCT). AAK and GF work with Dr. Idell and likewise have COI plans managed through UTHSCT and otherwise have no competing interests to declare. ROW III has received research support funding from LTI. All other authors have no competing interests related to the subject matter of this contribution.

## FUNDING

Grants: NIH NHLBI RO‐1HL118401 (MPI: SI Contact and Tyler Site PI; MRW – Temple Site PI; ROW – Austin Site PI; PE – Galveston Site PI), 1U54ES027698 (SI; Site PI, subcontract), TLL Temple Endowed Chair in Idiopathic Pulmonary Fibrosis, Texas Lung Injury Institute, NIH SMARTT (Science Moving towards Research Translation and Therapy) Contract No. HSN268201100014C (SI, PI); Shriners Hospital for Children 84050 (PE, PI).

## AUTHOR CONTRIBUTIONS

Marla R. Wolfson, Perenlei Enkhbaatar, Robert O. Williams, III, Andrey A. Komissarov, Galina Florova, Steven I. Idell, and Thomas H. Shaffer contributed to the study design, experiments, and manuscript. Satoshi Fukuda contributed to the study design and experiments. Christina. L. Nelson, Soraya Hengsawas Surasarang, S. Sahakijpijarn, Gennaro Calendo, and Krishna Sarva performed experiments.

## DATA SHARING AND DATA ACCESSIBILITY

All authors confirm adherence to the policy. The data that support the findings of this study are openly available at https://figshare.com.

## ETHICS APPROVAL

All animal studies were approved by the Institutional Animal Care and Use Committees of The University of Texas Medical Branch at Galveston and The University of Texas Health Science Center at Tyler. Animals were managed according to the Guiding Principles in the Care and Use of Animals of the National Institutes of Health.

## Supporting information

Supporting Figure S1

Supporting Figure S2

Supporting Table S1

Supporting Table S2

Supporting Table S3

Supporting Table S4

## References

[1] Herndon DN , Traber LD , Linares H , et al. Etiology of the pulmonary pathophysiology associated with inhalation injury. Resuscitation. 1986;14:43‐59. 3024279 10.1016/0300-9572(86)90006-7

[2] Herndon DN , Traber DL , Niehaus GD , Linares HA , Traber LD . The pathophysiology of smoke inhalation injury in a sheep model. J Trauma. 1984;24:1044‐1051. 6512897 10.1097/00005373-198412000-00007

[3] Enkhbaatar P , PruittBA Jr , Suman O , et al. Pathophysiology, research challenges, and clinical management of smoke inhalation injury. Lancet. 2016;388:1437‐1446. 27707500 10.1016/S0140-6736(16)31458-1 PMC5241273

[4] Enkhbaatar P , Traber DL . Pathophysiology of acute lung injury in combined burn and smoke inhalation injury. Clin Sci (Lond). 2004;107:137‐143. 15151496 10.1042/CS20040135

[5] Toon MH , Maybauer MO , Greenwood JE , Maybauer DM , Fraser JF . Management of acute smoke inhalation injury. Crit Care Resusc. 2010;12:53‐61. 20196715

[6] Miller AC , Elamin EM , Suffredini AF . Inhaled anticoagulation regimens for the treatment of smoke inhalation‐associated acute lung injury: a systematic review. Crit Care Med. 2014;42:413‐419. 24158173 10.1097/CCM.0b013e3182a645e5 PMC3947059

[7] Fukuda S , Enkhbaatar P , Nelson C , et al. Lack of durable protection against cotton smoke‐induced acute lung injury in sheep by nebulized single chain urokinase plasminogen activator or tissue plasminogen activator. Clin Trans Med. 2018;7:17. 10.1186/s40169-018-0196-3 PMC6006005 29916009

[8] Komissarov AA , Mazar AP , Koenig K , Kurdowska AK , Idell S . Regulation of intrapleural fibrinolysis by urokinase‐alpha‐macroglobulin complexes in tetracycline‐induced pleural injury in rabbits. Am J Physiol Lung Cell Mol Physiol. 2009;297:L568‐L577. 19666776 10.1152/ajplung.00066.2009 PMC2770793

[9] Wolfson MR , Greenspan JS , Shaffer TH . Pulmonary administration of vasoactive substances by perfluorochemical ventilation. Pediatrics. 1996;97:449‐455. 8632927

[10] Kazzaz JA , Strayer MS , Wu J , et al. Perfluorochemical liquid‐adenovirus suspensions enhance gene delivery to the distal lung. Pulm Med. 2011;2011:918036. 21876799 10.1155/2011/918036 PMC3159382

[11] Li JT , Bonneau LA , Zimmerman JJ , Weiss DJ . Perfluorochemical (PFC) liquid enhances recombinant adenovirus vector‐mediated viral interleukin‐10 (AdvIL‐10) expression in rodent lung. J Inflamm (Lond). 2007;4:9. 17472748 10.1186/1476-9255-4-9 PMC1868755

[12] Cox CA , Cullen AB , Wolfson MR , Shaffer TH . Intratracheal administration of perfluorochemical‐gentamicin suspension: a comparison to intravenous administration in normal and injured lungs. Pediatr Pulmonol. 2001;32:142‐151. 11477731 10.1002/ppul.1100

[13] Zelinka MA , Wolfson MR , Calligaro S , Rubenstein SD , Greenspan JS , Shaffer TH . A comparison of intratracheal and intravenous administration of gentamicin during liquid ventilation. Eur J Pediatr. 1996;156:401‐404. 10.1007/s004310050625 9177987

[14] Sarafidis K , Malone DJ , Zhu G , et al. Perfluorochemical augmented rhSOD delivery attenuates inflammation in the immature lung. J Neonatal Perinatal Med. 2008;1:159‐168.

[15] Lisby DA , Ballard PL , Fox WW , Wolfson MR , Shaffer TH , Gonzales LW . Enhanced distribution of adenovirus‐mediated gene transfer to lung parenchyma by perfluorochemical liquid. Hum Gene Ther. 1997;8:919‐928. 9195214 10.1089/hum.1997.8.8-919

[16] Chappell SE , Wolfson MR , Shaffer TH . A comparison of surfactant delivery with conventional mechanical ventilation and partial liquid ventilation in meconium aspiration injury. Respir Med. 2001;95:612‐617. 11453320 10.1053/rmed.2001.1114

[17] Wolfson MR , Shaffer TH . Liquid ventilation: an adjunct for respiratory management. Paediatr Anaesth. 2004;14:15‐23. 14717869 10.1046/j.1460-9592.2003.01206.x

[18] Wolfson MR , Hirschl RB , Jackson JC , et al. Multicenter comparative study of conventional mechanical gas ventilation to tidal liquid ventilation in oleic acid injured sheep. ASAIO J. 2008;54:256‐269. 18496275 10.1097/MAT.0b013e318168fef0

[19] Fitzpatrick JC , Jordan BS , Salman N , Williams J . The use of perfluorocarbon‐associated gas exchange to improve ventilation and decrease mortality after inhalation injury in a neonatal swine model. J Pediatr Surg. 1997;32:192‐196. 9044120 10.1016/s0022-3468(97)90177-9

[20] Colton DM , Till GO , Johnson KJ , Dean SB , Bartlett RH , Hirschl RB . Neutrophil accumulation is reduced during partial liquid ventilation. Crit Care Med. 1998;26:1716‐1724. 9781730 10.1097/00003246-199810000-00028

[21] Greenspan JS , Wolfson MR , Rubenstein SD , Shaffer TH . Liquid ventilation of human preterm neonates. J Pediatr. 1990;117:106‐111. 2115078 10.1016/s0022-3476(05)82457-6

[22] Leach CL , Greenspan JS , Rubenstein SD , et al. Partial liquid ventilation with perflubron in premature infants with severe respiratory distress syndrome. The LiquiVent Study Group. N Engl J Med. 1996;335:761‐767. 8778584 10.1056/NEJM199609123351101

[23] Hirschl RB , Croce M , Gore D , et al. Prospective, randomized, controlled pilot study of partial liquid ventilation in adult acute respiratory distress syndrome. Am J Respir Crit Care Med. 2002;165:781‐787. 11897644 10.1164/ajrccm.165.6.2003052

[24] Ding H , Lv Q , Wu S , et al. Intratracheal instillation of perfluorohexane modulates the pulmonary immune microenvironment by attenuating early inflammatory factors in patients with smoke inhalation injury: a randomized controlled clinical trial. J Burn Care Res. 2017;38:251‐259. 28099236 10.1097/BCR.0000000000000496

[25] Enkhbaatar P , Cox RA , Traber LD , et al. Aerosolized anticoagulants ameliorate acute lung injury in sheep after exposure to burn and smoke inhalation. Crit Care Med. 2007;35:2805‐2810. 18074480 10.1097/01.ccm.0000291647.18329.83

[26] Enkhbaatar P , Murakami K , Cox R , et al. Aerosolized tissue plasminogen inhibitor improves pulmonary function in sheep with burn and smoke inhalation. Shock. 2004;22:70‐75. 15201705 10.1097/01.shk.0000129201.38588.85

[27] Cox RA , Burke AS , Oliveras G , et al. Acute bronchial obstruction in sheep: histopathology and gland cytokine expression. Exp Lung Res. 2005;31:819‐837. 16684715 10.1080/01902140600574967

[28] Ortiz RM , Cilley RE , Bartlett RH . Extracorporeal membrane oxygenation in pediatric respiratory failure. Pediatr Clin North Am. 1987;34:39‐46. 3808772 10.1016/s0031-3955(16)36179-x

[29] Notter RH , Egan EA , Kwong MS , Holm BA , Shapiro DL . Lung surfactant replacement in premature lambs with extracted lipids from bovine lung lavage: effects of dose, dispersion technique, and gestational age. Pediatr Res. 1985;19:569‐577. 3839302 10.1203/00006450-198506000-00014

[30] Surasarang SH , Sahakijpijarn S , Florova G , et al. Nebulization of single‐chain tissue‐type and single‐chain urokinase plasminogen activator for treatment of inhalational smoke‐induced acute lung injury. J Drug Deliv Sci Technol. 2018;48:19‐27. 30123328 10.1016/j.jddst.2018.04.013 PMC6095669

[31] Wolfson MR , Greenspan JS , Deoras KS , Rubenstein SD , Shaffer TH . Comparison of gas and liquid ventilation: clinical, physiological, and histological correlates. J Appl Physiol. 1992;72:1024‐1031. 1568956 10.1152/jappl.1992.72.3.1024

[32] Wolfson MR , Wu J , Hubert TL , Gregory TJ , Mazela J , Shaffer TH . Lucinactant attenuates pulmonary inflammatory response, preserves lung structure, and improves physiologic outcomes in a preterm lamb model of RDS. Pediatr Res. 2012;72:375‐383. 22821059 10.1038/pr.2012.96 PMC3888789

[33] Deoras KS , Wolfson MR , Searls RL , Hilfer SR , Sheffield JB , Shaffer TH . Use of a touch sensitive screen and computer assisted image analysis for quantitation of developmental changes in pulmonary structure. Pediatr Pulmonol. 1990;9:109‐118. 2399044 10.1002/ppul.1950090208

[34] Munoz‐Barrutia A , Ceresa M , Artaechevarria X , Montuenga LM , Ortiz‐de‐Solorzano C . Quantification of lung damage in an elastase‐induced mouse model of emphysema. Int J Biomed Imaging. 2012;2012:734734. 23197972 10.1155/2012/734734 PMC3503307

[35] Jacob RE , Carson JP , Gideon KM , Amidan BG , Smith CL , Lee KM . Comparison of two quantitative methods of discerning airspace enlargement in smoke‐exposed mice. PLoS One. 2009;4:e6670. 19688093 10.1371/journal.pone.0006670 PMC2722737

[36] Parameswaran H , Majumdar A , Ito S , Alencar AM , Suki B . Quantitative characterization of airspace enlargement in emphysema. J Appl Physiol. 1985;100:186‐193. 10.1152/japplphysiol.00424.2005 16166240

[37] Sallon C , Soulet D , Provost PR , Tremblay Y . Automated high‐performance analysis of lung morphometry. Am J Respir Cell Mol Biol. 2015;53:149‐158. 25695836 10.1165/rcmb.2014-0469MA

[38] Cox RA , Burke AS , Soejima K , et al. Airway obstruction in sheep with burn and smoke inhalation injuries. Am J Respir Cell Mol Biol. 2003;29:295‐302. 12936906 10.1165/rcmb.4860

[39] Schindelin J , Arganda‐Carreras I , Frise E , et al. Fiji: an open‐source platform for biological‐image analysis. Nat Methods. 2012;9:676‐682. 22743772 10.1038/nmeth.2019 PMC3855844

[40] Komissarov AA , Florova G , Azghani AO , et al. Dose dependency of outcomes of intrapleural fibrinolytic therapy in new rabbit empyema models. Am J Physiol Lung Cell Mol Physiol. 2016;311:L389‐L399. 27343192 10.1152/ajplung.00171.2016 PMC5142452

[41] Komissarov AA , Florova G , Idell S . Effects of extracellular DNA on plasminogen activation and fibrinolysis. J Biol Chem. 2011;286:41949‐41962. 21976662 10.1074/jbc.M111.301218 PMC3234961

[42] American Academy of Pediatrics . Reducing the number of deaths and injuries from residential fires. Pediatrics. 2000;105:1355‐1357. 10835082 10.1542/peds.105.6.1355

[43] Tuinman PR , Dixon B , Levi M , Juffermans NP , Schultz MJ . Nebulized anticoagulants for acute lung injury — a systematic review of preclinical and clinical investigations. Crit Care. 2012;16:R70. 22546487 10.1186/cc11325 PMC3681399

[44] O'Donnell J . Anticoagulants: therapeutics, risks, and toxicity–special emphasis on heparin‐induced thrombocytopenia (HIT). J Pharm Pract. 2012;25:22‐29. 22491766 10.1177/0897190011431146

[45] Juschten J , Tuinman PR , Juffermans NP , Dixon B , Levi M , Schultz MJ . Nebulized anticoagulants in lung injury in critically ill patients—an updated systematic review of preclinical and clinical studies. Ann Transl Med. 2017;5:444. 29264361 10.21037/atm.2017.08.23 PMC5721225

[46] Phua J , Badia JR , Adhikari NK , et al. Has mortality from acute respiratory distress syndrome decreased over time?: a systematic review. Am J Respir Crit Care Med. 2009;179:220‐227. 19011152 10.1164/rccm.200805-722OC

[47] Komissarov AA , Stankowska D , Krupa A , et al. Novel aspects of urokinase function in the injured lung: role of alpha2‐macroglobulin. Am J Physiol Lung Cell Mol Physiol. 2012;303:L1037‐L1045. 23064953 10.1152/ajplung.00117.2012 PMC3532585

[48] Wolfson MR , Stern RG , Kechner N , Sekins KM , Shaffer TH . Utility of a perfluorochemical liquid for pulmonary diagnostic imaging. Artif Cells Blood Substit Immobil Biotechnol. 1994;22:1409‐1420. 7849952 10.3109/10731199409138845

[49] Shaffer TH , Tran N , Bhutani VK , Sivieri EM . Cardiopulmonary function in very preterm lambs during liquid ventilation. Pediatr Res. 1983;17:680‐684. 6889011 10.1203/00006450-198308000-00016

[50] Wolfson MR , Tran N , Bhutani VK , Shaffer TH . A new experimental approach for the study of cardiopulmonary physiology during early development. J Appl Physiol. 1988;65:1436‐1443. 3182511 10.1152/jappl.1988.65.3.1436

[51] Wolfson MR , Kechner NE , Roache RF , et al. Perfluorochemical rescue after surfactant treatment: effect of perflubron dose and ventilatory frequency. J Appl Physiol. 1998;84:624‐640. 9475875 10.1152/jappl.1998.84.2.624

[52] Wolfson MR , Shaffer TH . Pulmonary applications of perfluorochemical liquids: ventilation and beyond. Paediatr Respir Rev. 2005;6:117‐127. 15911457 10.1016/j.prrv.2005.03.010

[53] Lozano JA , Castro JA , Rodrigo I . Partial liquid ventilation with perfluorocarbons for treatment of ARDS in burns. Burns. 2001;27:635‐642. 11525860 10.1016/s0305-4179(01)00010-9

[54] Cindrick LL , Gore DC , Herndon DN , Traber LD , Traber DL . Bronchoscopic lavage with perfluorocarbon decreases postprocedure hypoxemia in an ovine model of smoke inhalation. J Trauma. 1999;46:129‐135. 9932695 10.1097/00005373-199901000-00022

[55] Dickson EW , Sivilotti ML , Mangolds G , et al. Core rewarming via warm lavage liquid ventilation in a swine model of hypothermia‐associated ventricular fibrillation. Acad Emerg Med. 2001;8:82‐84. 11136157 10.1111/j.1553-2712.2001.tb00561.x

