# Supplementary figures and images for: Perfluorochemical‐facilitated plasminogen activator delivery to the airways: A novel treatment for inhalational smoke‐induced acute lung injury
Source: Clin Transl Med. 2020 Apr 30;10(1):258–74. doi: 10.1002/ctm2.26 (PMC7240845; doi:10.1002/ctm2.26)

Supplementary Figure 1


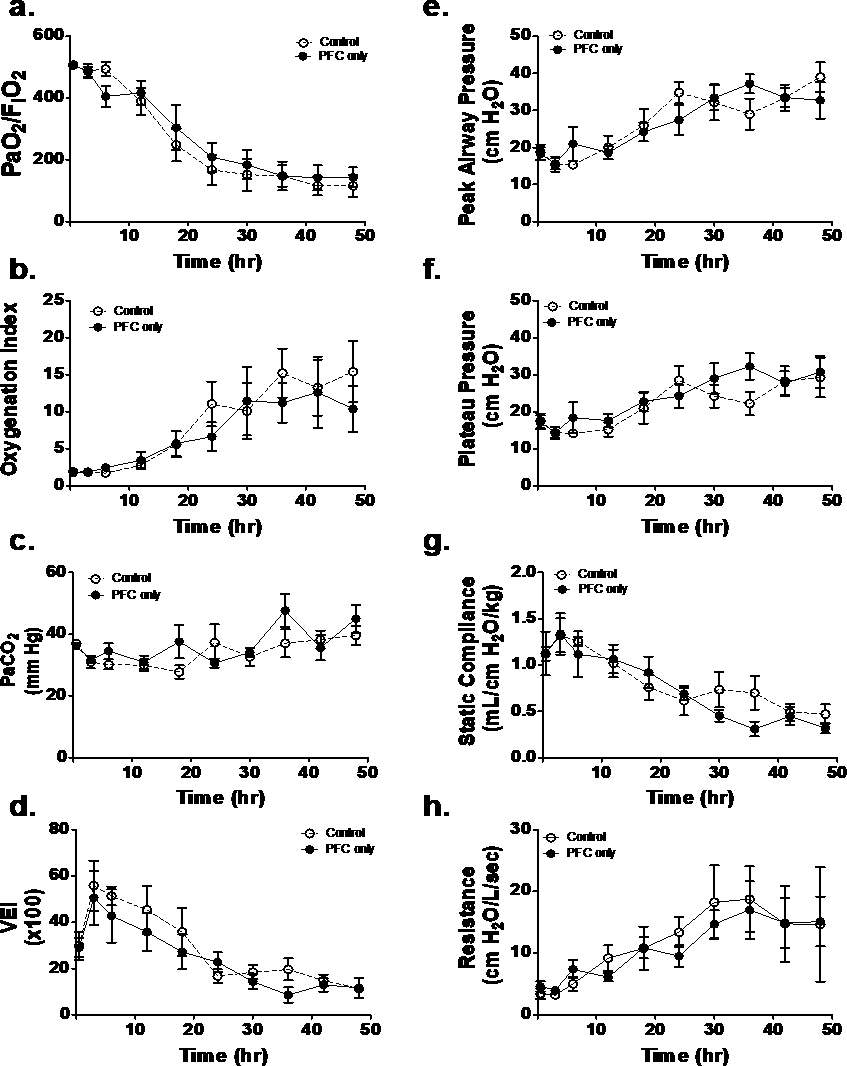

Supplement: Supplementary file 1 — Supporting Figure S1 [file CTM2-10-258-s001.docx]

Supplementary Figure 2


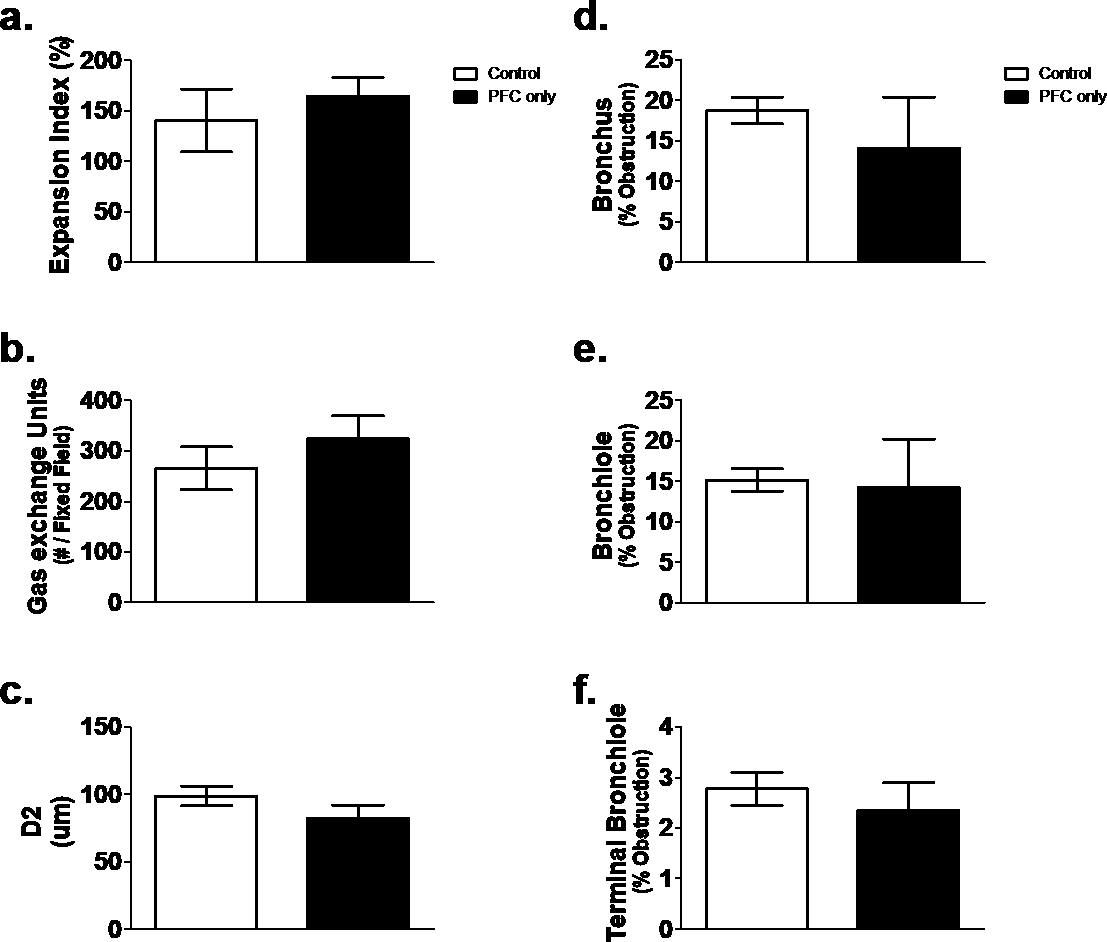

Supplement: Supplementary file 2 — Supporting Figure S2 [file CTM2-10-258-s002.docx]
