# Supplementary material for: Perfluorochemical‐facilitated plasminogen activator delivery to the airways: A novel treatment for inhalational smoke‐induced acute lung injury
Source: Clin Transl Med. 2020 Apr 30;10(1):258–74. doi: 10.1002/ctm2.26 (PMC7240845; doi:10.1002/ctm2.26)
Supplement: Supplementary file 3 — Supporting Table S1 [file CTM2-10-258-s003.docx]

**Supplemental Table 1.** Lyophilized cycle.

| **Step** | **Mode** | **Rate (°C/min)** | **Temperature (°C)** | **Pressure (mTorr)** | **Time (min)** |
| --- | --- | --- | --- | --- | --- |
| Load | Hold | - | 5 | - | 60 |
| Freeze | Ramp | 0.5 | -55 | - | 120 |
|  | Hold | - | -55 | - | 120 |
| Anneal | Ramp | 0.5 | -15 | - | 80 |
|  | Hold | - | -15 | - | 120 |
| Freeze | Ramp | 0.5 | -55 | - | 80 |
|  | Hold | - | -55 | - | 240 |
| Evacuate | Hold | - | -55 | 100 | 30 |
| Primary drying | Ramp | 0.1 | -30 | 100 | 250 |
|  | Hold | - | -30 | 100 | 660 |
| Secondary drying | Ramp | 0.08 | 30 | 100 | 720 |
|  | Hold | - | 30 | 100 | 240 |
