# Supplementary material for: Perfluorochemical‐facilitated plasminogen activator delivery to the airways: A novel treatment for inhalational smoke‐induced acute lung injury
Source: Clin Transl Med. 2020 Apr 30;10(1):258–74. doi: 10.1002/ctm2.26 (PMC7240845; doi:10.1002/ctm2.26)
Supplement: Supplementary file 4 — Supporting Table S2 [file CTM2-10-258-s004.docx]

**Supplemental Table 2.** The specific enzyme activity of plasminogen activator/perfluorochemical suspensions after storage.

| **Storage temperature**  **(°C)** | **Time point (hours)** | **scuPA (%)** | **tPA (%)** |
| --- | --- | --- | --- |
| - | 0 | 50.6 ± 2.1 | 77.0 ± 6.7 |
| 6-10 °C | 24 | 51.2 ± 2.3 | 76.5 ± 6.9 |
|  | 48 | 53.8 ± 3.3 | 80.8 ± 4.7 |
| 25 °C | 3 | 50.0 ± 0.4 | 76.0 ± 9.5 |
