# Supplementary material for: Perfluorochemical‐facilitated plasminogen activator delivery to the airways: A novel treatment for inhalational smoke‐induced acute lung injury
Source: Clin Transl Med. 2020 Apr 30;10(1):258–74. doi: 10.1002/ctm2.26 (PMC7240845; doi:10.1002/ctm2.26)
Supplement: Supplementary file 5 — Supporting Table S3 [file CTM2-10-258-s005.docx]

**Supplemental Table 3.** Ventilatory parameters.

A. Tidal volume (VT) expressed as set on ventilator (VT set), actual (VT actual) as measured by the ventilator, and as calculated effective VT, as VT actual as percentage of VT set. tPA: tissue plasminogen activator; scuPA: single chain urokinase plasminogen activator; PFC: perfluorochemical.

| **V_T_ set** | **PFC Only** | | **tPA 4 mg in PFC** | | **tPA 8 mg in PFC** | | **scuPA 4 mg in PFC** | | **scuPA 8 mg in PFC** | |
| --- | --- | --- | --- | --- | --- | --- | --- | --- | --- | --- |
| **mL/kg** | **mean** | **SEM** | **mean** | **SEM** | **mean** | **SEM** | **mean** | **SEM** | **mean** | **SEM** |
| Baseline | 12.5 | 7.0 | 11.1 | 0.5 | 12.1 | 0.4 | 12.7 | 0.5 | 12.2 | 0.2 |
| 3 hrs | 12.5 | 7.0 | 11.1 | 0.5 | 12.1 | 0.4 | 12.7 | 0.5 | 12.2 | 0.2 |
| 6 hrs | 12.5 | 7.0 | 11.1 | 0.5 | 12.1 | 0.4 | 12.7 | 0.5 | 12.2 | 0.2 |
| 12 hrs | 12.5 | 7.0 | 11.1 | 0.5 | 12.1 | 0.4 | 12.7 | 0.5 | 12.2 | 0.2 |
| 18 hrs | 12.5 | 7.0 | 11.1 | 0.5 | 12.1 | 0.4 | 12.7 | 0.5 | 12.2 | 0.2 |
| 24 hrs | 12.5 | 7.0 | 11.1 | 0.5 | 12.1 | 0.4 | 12.7 | 0.5 | 12.2 | 0.2 |
| 30 hrs | 12.5 | 7.0 | 11.1 | 0.5 | 12.1 | 0.4 | 12.7 | 0.5 | 12.2 | 0.2 |
| 36 hrs | 12.5 | 7.0 | 11.1 | 0.5 | 12.1 | 0.4 | 10.5 | 2.2 | 12.0 | 0.2 |
| 42 hrs | 12.3 | 5.7 | 11.1 | 0.5 | 12.1 | 0.4 | 10.5 | 2.2 | 12.0 | 0.2 |
| 48 hrs | 12.3 | 5.7 | 11.1 | 0.5 | 12.1 | 0.4 | 10.5 | 2.2 | 12.0 | 0.2 |
|  |  |  |  |  |  |  |  |  |  |  |
| **V_T_ actual** | **PFC Only** | | **tPA 4 mg in PFC** | | **tPA 8 mg in PFC** | | **scuPA 4 mg in PFC** | | **scuPA 8 mg in PFC** | |
| **mL/kg** | **mean** | **SEM** | **mean** | **SEM** | **mean** | **SEM** | **mean** | **SEM** | **mean** | **SEM** |
| Baseline | 11.3 | 0.6 | 9.4 | 1.9 | 12.2 | 0.3 | 12.8 | 0.6 | 12.4 | 0.3 |
| 3 hrs | 12.8 | 0.7 | 10.9 | 0.5 | 12.1 | 0.4 | 13.5 | 0.7 | 12.8 | 0.2 |
| 6 hrs | 12.9 | 0.6 | 11.3 | 0.4 | 12.2 | 0.4 | 13.1 | 0.4 | 11.9 | 0.3 |
| 12 hrs | 13.1 | 0.4 | 11.4 | 0.5 | 11.9 | 0.1 | 13.0 | 0.4 | 12.3 | 0.2 |
| 18 hrs | 12.9 | 0.5 | 11.0 | 0.7 | 11.7 | 0.4 | 12.5 | 0.6 | 12.8 | 0.3 |
| 24 hrs | 12.1 | 0.3 | 11.2 | 0.7 | 12.1 | 0.4 | 12.6 | 0.7 | 11.6 | 0.6 |
| 30 hrs | 11.8 | 0.3 | 10.3 | 0.5 | 12.0 | 0.4 | 12.5 | 0.7 | 11.8 | 0.4 |
| 36 hrs | 9.4 | 1.4 | 10.2 | 0.5 | 11.7 | 0.7 | 11.3 | 2.0 | 11.9 | 0.5 |
| 42 hrs | 9.9 | 1.0 | 10.8 | 0.7 | 11.0 | 0.7 | 11.4 | 2.1 | 11.6 | 0.2 |
| 48 hrs | 8.2 | 1.1 | 9.7 | 0.7 | 10.4 | 0.8 | 12.0 | 2.2 | 11.6 | 0.5 |
|  |  |  |  |  |  |  |  |  |  |  |
| **Effective V_T_** | **PFC Only** | | **tPA 4 mg in PFC** | | **tPA 8 mg in PFC** | | **scuPA 4 mg in PFC** | | **scuPA 8 mg in PFC** | |
| **(act/set %)** | **mean** | **SEM** | **mean** | **SEM** | **mean** | **SEM** | **mean** | **SEM** | **mean** | **SEM** |
| Baseline | 93.4 | 5.9 | 99.2 | 1.7 | 100.7 | 1.6 | 100.8 | 1.9 | 101.8 | 2.6 |
| 3 hrs | 103.0 | 7.6 | 99.0 | 1.3 | 99.8 | 2.1 | 107.4 | 5.5 | 105.6 | 2.1 |
| 6 hrs | 103.4 | 6.0 | 102.2 | 1.1 | 101.3 | 2.2 | 103.9 | 2.0 | 98.5 | 4.1 |
| 12 hrs | 105.1 | 3.9 | 103.5 | 2.0 | 99.0 | 2.4 | 102.8 | 2.3 | 101.6 | 3.2 |
| 18 hrs | 103.6 | 3.4 | 98.9 | 2.7 | 96.9 | 2.5 | 98.9 | 1.5 | 105.6‡¥ | 3.4 |
| 24 hrs | 97.5 | 3.5 | 101.1† | 2.7 | 100.7† | 4.0 | 99.3 | 3.9 | 95.4 | 5.7 |
| 30 hrs | 94.7 | 2.9 | 93.3 | 3.4 | 99.1† | 2.9 | 99.1 | 4.8 | 97.7 | 4.6 |
| 36 hrs | 75.2** | 11.0 | 92.8† | 3.0 | 96.8† | 6.2 | 89.7† | 3.7 | 100.9††‡ | 6.5 |
| 42 hrs | 84.0 | 9.1 | 97.7† | 2.9 | 90.7† | 4.8 | 89.7 | 4.7 | 97.4†‡¥ | 3.0 |
| 48 hrs | 66.5** | 9.3 | 88.2† | 5.6 | 86.5† | 6.6 | 94.5†† | 6.2 | 96.1††¥ | 5.6 |

*p < 0.05, **p < 0.01 vs. Baseline; †p < 0.05, ††p < 0.01 vs. PFC only; ‡p < 0.05, ‡‡p < 0.01 vs. Dose; ¥ p < 0.05; ¥¥ p < 0.01 vs. plasminogen activator type.

B. Breathing Frequency (Freq) expressed as set on ventilator (Freq set), actual (Freq actual) as 720 measured by the ventilator, and as calculated effective Freq, as Freq actual as percentage of 721 Freq set. tPA: tissue plasminogen activator; scuPA: single chain urokinase plasminogen activator; PFC: perfluorochemical.

| **Freq set** | **PFC Only** | | **tPA 4 mg in PFC** | | **tPA 8 mg in PFC** | | **scuPA 4 mg in PFC** | | **scuPA 8 mg in PFC** | |
| --- | --- | --- | --- | --- | --- | --- | --- | --- | --- | --- |
| **br/min** | **mean** | **SEM** | **mean** | **SEM** | **mean** | **SEM** | **mean** | **SEM** | **mean** | **SEM** |
| Baseline | 20.0 | 0.0 | 20.0 | 0.0 | 20.0 | 0.0 | 20.0 | 0.0 | 20.0 | 0.0 |
| 3 hrs | 20.0 | 0.0 | 20.0 | 0.0 | 20.0 | 0.0 | 20.0 | 0.0 | 20.0 | 0.0 |
| 6 hrs | 20.0 | 1.3 | 20.0 | 0.0 | 20.0 | 0.0 | 20.0 | 0.0 | 20.0 | 0.0 |
| 12 hrs | 20.0 | 1.3 | 20.0 | 0.0 | 20.0 | 0.0 | 20.0 | 0.0 | 20.0 | 0.0 |
| 18 hrs | 21.0 | 2.1 | 20.0 | 0.0 | 19.3 | 0.4 | 20.0 | 0.0 | 20.0 | 0.0 |
| 24 hrs | 21.8 | 1.1 | 22.5 | 1.7 | 19.3 | 0.4 | 20.0 | 0.0 | 20.0 | 0.0 |
| 30 hrs | 23.8 | 1.9 | 21.8 | 1.2 | 19.3 | 0.4 | 20.0 | 0.0 | 20.0 | 0.0 |
| 36 hrs | 25.4 | 3.7 | 21.8 | 1.2 | 19.3 | 0.4 | 19.2 | 0.8 | 20.0 | 0.0 |
| 42 hrs | 25.0 | 3.5 | 21.8 | 1.2 | 19.3 | 0.4 | 19.0 | 1.1 | 20.0 | 0.0 |
| 48 hrs | 23.8 | 2.4 | 23.2 | 1.5 | 25.2 | 4.4 | 19.0 | 1.1 | 20.0 | 0.0 |
|  |  |  |  |  |  |  |  |  |  |  |
| **Freq act** | **PFC Only** | | **tPA 4 mg in PFC** | | **tPA 8 mg in PFC** | | **scuPA 4 mg in PFC** | | **scuPA 8 mg in PFC** | |
| **br/min** | **mean** | **SEM** | **mean** | **SEM** | **mean** | **SEM** | **mean** | **SEM** | **mean** | **SEM** |
| Baseline | 32.2 | 3.7 | 25.3 | 1.8 | 26.7 | 3.2 | 27.3 | 3.5 | 28.8 | 2.7 |
| 3 hrs | 28.4 | 3.4 | 34.8 | 1.6 | 31.0 | 3.1 | 35.2 | 2.6 | 29.2 | 4.1 |
| 6 hrs | 26.6 | 5.8 | 24.7 | 2.9 | 24.0 | 2.4 | 28.7 | 2.0 | 23.5 | 2.3 |
| 12 hrs | 26.2 | 3.8 | 22.2 | 1.4 | 23.3 | 1.1 | 26.0 | 1.9 | 23.0 | 0.9 |
| 18 hrs | 21.6 | 0.8 | 27.3 | 1.7 | 22.3 | 1.3 | 26.7 | 2.9 | 24.7 | 2.4 |
| 24 hrs | 31.2 | 6.1 | 27.8 | 3.4 | 24.2 | 3.2 | 22.0 | 1.2 | 30.7 | 3.4 |
| 30 hrs | 32.6 | 3.7 | 26.7 | 2.7 | 24.0 | 2.0 | 27.2 | 1.9 | 28.5 | 3.5 |
| 36 hrs | 46.0 | 9.5 | 26.3 | 2.4 | 19.8 | 0.7 | 31.7 | 2.7 | 20.8 | 0.4 |
| 42 hrs | 35.0 | 5.9 | 29.2 | 2.1 | 20.8 | 0.8 | 26.8 | 5.4 | 20.2 | 0.2 |
| 48 hrs | 40.3 | 8.6 | 27.3 | 2.5 | 31.8 | 7.7 | 26.8 | 5.7 | 20.8 | 0.6 |
|  |  |  |  |  |  |  |  |  |  |  |
| **Effective Freq** | **PFC Only** | | **tPA 4 mg in PFC** | | **tPA 8 mg in PFC** | | **scuPA 4 mg in PFC** | | **scuPA 8 mg in PFC** | |
| **(act/set %)** | **mean** | **SEM** | **mean** | **SEM** | **mean** | **SEM** | **mean** | **SEM** | **mean** | **SEM** |
| Baseline | 161.0 | 18.5 | 126.7 | 8.8 | 133.3 | 16.2 | 136.5 | 17.4 | 144.2 | 13.6 |
| 3 hrs | 142.0 | 16.9 | 174.2 | 7.8 | 155.0 | 15.6 | 175.8 | 13.2 | 145.8 | 20.5 |
| 6 hrs | 129.1 | 19.9 | 123.3 | 14.5 | 120.0 | 11.8 | 143.3 | 10.0 | 117.5‡ | 11.7 |
| 12 hrs | 129.7 | 12.0 | 110.8† | 7.0 | 116.7† | 5.6 | 130.0 | 9.3 | 115.0 | 4.7 |
| 18 hrs | 106.3 | 10.1 | 136.7† | 8.4 | 115.1† | 4.8 | 133.3 | 14.7 | 123.3 | 12.2 |
| 24 hrs | 139.4 | 20.0 | 123.6† | 11.3 | 124.2† | 14.6 | 110† | 5.8 | 153.3‡ | 17.0 |
| 30 hrs | 139.2 | 17.7 | 123.5† | 14.4 | 123.3† | 8.3 | 135.8 | 9.7 | 142.5 | 17.6 |
| 36 hrs | 184.9 | 37.8 | 121.5†† | 11.7 | 102.5†† | 2.5 | 154.8† | 9.2 | 104††‡ | 1.9 |
| 42 hrs | 168.5 | 35.8 | 135.7† | 13.0 | 107.8†† | 3.5 | 139.7 | 24.8 | 101††‡ | 1.0 |
| 48 hrs | 169.9 | 34.3 | 117.1†† | 4.1 | 120.2† | 9.3 | 141.3 | 28.0 | 104††‡ | 2.9 |

*p < 0.05, **p < 0.01 vs. Baseline; †p < 0.05, ††p < 0.01 vs. PFC only; ‡p< 0.05, 726 ‡‡p < 0.01 vs. Dose; ¥ p < 0.05; ¥¥ p < 0.01 vs. plasminogen activator type.

C. Minute ventilation (MV) expressed as set on ventilator (MV set), actual (MV actual) as calculated using frequency and tidal volume measurements from the ventilator, and as calculated effective MV, as MV actual as percentage of MV set. tPA: tissue plasminogen activator; scuPA: single chain urokinase plasminogen activator; PFC: perfluorochemical.

| **MV set** | **PFC Only** | | **tPA 4 mg in PFC** | | **tPA 8 mg in PFC** | | **scuPA 4 mg in PFC** | | **scuPA 8 mg in PFC** | |
| --- | --- | --- | --- | --- | --- | --- | --- | --- | --- | --- |
| **mL/min/kg** | **mean** | **SEM** | **mean** | **SEM** | **mean** | **SEM** | **mean** | **SEM** | **mean** | **SEM** |
| Baseline | 249.6 | 4.7 | 221.1 | 10.1 | 242.0 | 7.4 | 343.5 | 42.6 | 243.1 | 4.7 |
| 3 hrs | 249.6 | 4.7 | 221.1 | 10.1 | 242.0 | 7.4 | 447.0 | 42.3 | 243.1 | 4.7 |
| 6 hrs | 249.3 | 15.3 | 221.1 | 10.1 | 242.0 | 7.4 | 365.3 | 33.9 | 243.1 | 4.7 |
| 12 hrs | 249.3 | 15.3 | 221.1 | 10.1 | 242.0 | 7.4 | 330.7 | 31.1 | 243.1 | 4.7 |
| 18 hrs | 261.7 | 26.2 | 221.1 | 10.1 | 233.5 | 6.0 | 342.6 | 49.0 | 243.1 | 4.7 |
| 24 hrs | 272.7 | 17.8 | 244.7 | 8.0 | 233.5 | 6.0 | 279.6 | 21.2 | 243.1 | 4.7 |
| 30 hrs | 298.7 | 29.0 | 238.4 | 2.0 | 233.5 | 6.0 | 341.3 | 21.2 | 243.1 | 4.7 |
| 36 hrs | 320.0 | 53.5 | 238.4 | 2.0 | 233.5 | 6.0 | 310.5 | 65.9 | 240.0 | 4.4 |
| 42 hrs | 308.5 | 45.9 | 238.4 | 2.0 | 233.5 | 6.0 | 274.1 | 70.4 | 240.0 | 4.4 |
| 48 hrs | 293.0 | 32.4 | 254.4 | 16.4 | 303.0 | 51.6 | 276.5 | 74.4 | 240.0 | 4.4 |
|  |  |  |  |  |  |  |  |  |  |  |
| **MV act** | **PFC Only** | | **tPA 4 mg in PFC** | | **tPA 8 mg in PFC** | | **scuPA 4 mg in PFC** | | **scuPA 8 mg in PFC** | |
| **mL/min/kg** | **mean** | **SEM** | **mean** | **SEM** | **mean** | **SEM** | **mean** | **SEM** | **mean** | **SEM** |
| Baseline | 371.8 | 43.3 | 298.6 | 27.4 | 320.9 | 32.4 | 347.2 | 44.8 | 356.2 | 33.2 |
| 3 hrs | 361.2 | 41.6 | 380.6 | 22.4 | 372.7 | 37.8 | 475.2 | 41.1 | 373.9 | 52.7 |
| 6 hrs | 343.8 | 76.1 | 273.3 | 21.7 | 292.0 | 25.5 | 379.6 | 36.9 | 279.8 | 28.2 |
| 12 hrs | 340.4 | 43.6 | 254.5 | 23.4 | 278.8 | 15.1 | 337.7 | 27.2 | 283.6 | 14.1 |
| 18 hrs | 279.3 | 14.5 | 296.9 | 20.7 | 261.1 | 15.9 | 339.9 | 50.0 | 314.1 | 27.1 |
| 24 hrs | 374.2 | 67.7 | 302.5 | 22.6 | 289.6 | 31.7 | 278.3 | 24.7 | 362.0 | 57.4 |
| 30 hrs | 387.5 | 51.0 | 275.5 | 35.4 | 283.3 | 16.6 | 338.1 | 25.7 | 336.7 | 41.9 |
| 36 hrs | 410.2 | 106.0 | 268.7 | 26.5 | 230.1 | 12.0 | 330.8 | 13.5 | 250.4 | 15.2 |
| 42 hrs | 343.6 | 25.8 | 318.6 | 36.9 | 226.6 | 10.2 | 286.4 | 30.7 | 233.3 | 3.1 |
| 48 hrs | 302.7 | 36.2 | 257.4 | 9.7 | 304.9 | 48.2 | 301.2 | 36.3 | 238.4 | 12.3 |
|  |  |  |  |  |  |  |  |  |  |  |
| **Effective MV** | **PFC Only** | | **tPA 4 mg in PFC** | | **tPA 8 mg in PFC** | | **scuPA 4 mg in PFC** | | **scuPA 8 mg in PFC** | |
| **(act/set %)** | **mean** | **SEM** | **mean** | **SEM** | **mean** | **SEM** | **mean** | **SEM** | **mean** | **SEM** |
| Baseline | 148.9 | 17.5 | 109.4 | 23.4 | 134.8 | 17.2 | 100.8 | 1.9 | 146.4 | 13.4 |
| 3 hrs | 145.0 | 17.1 | 172.3* | 8.0 | 155.5 | 17.7 | 107.4†† | 5.5 | 154.4‡ | 22.8 |
| 6 hrs | 134.3 | 22.6 | 126.2 | 15.0 | 122.2 | 13.7 | 103.9†† | 2.0 | 116.1 | 13.8 |
| 12 hrs | 135.4 | 10.6 | 114.9† | 8.3 | 116.1† | 7.9 | 102.8†† | 2.3 | 117‡ | 6.5 |
| 18 hrs | 110.2 | 11.0 | 134.5 | 7.3 | 111.7 | 6.1 | 98.9 | 1.5 | 129‡¥ | 10.4 |
| 24 hrs | 133.8 | 15.3 | 124.5 | 10.8 | 125.5 | 16.1 | 99.3† | 3.9 | 148.6‡¥ | 23.5 |
| 30 hrs | 133.1 | 20.0 | 115.6† | 14.7 | 121.3† | 5.9 | 99.1† | 4.8 | 139.4‡¥ | 18.6 |
| 36 hrs | 124.9 | 16.7 | 112.8† | 11.2 | 99.3† | 7.0 | 89.7† | 3.7 | 105.9†‡¥ | 7.0 |
| 42 hrs | 117.3 | 16.9 | 134.0 | 16.1 | 97.5† | 5.7 | 89.7† | 4.7 | 98.6†‡ | 1.9 |
| 48 hrs | 103.5 | 7.2 | 102.6 | 6.1 | 101.7 | 5.8 | 94.5 | 6.2 | 100.8 | 5.8 |

*p < 0.05, **p < 0.01 vs. Baseline; †p < 0.05, ††p < 0.01 vs. PFC only; ‡p< 0.05, ‡‡p < 0.01 vs. Dose; ¥ p < 0.05; ¥¥ p < 0.01 vs. plasminogen activator type.
