# Supplementary material for: Perfluorochemical‐facilitated plasminogen activator delivery to the airways: A novel treatment for inhalational smoke‐induced acute lung injury
Source: Clin Transl Med. 2020 Apr 30;10(1):258–74. doi: 10.1002/ctm2.26 (PMC7240845; doi:10.1002/ctm2.26)
Supplement: Supplementary file 6 — Supporting Table S4 [file CTM2-10-258-s006.docx]

**Supplemental Table 4.** Hemodynamic parameters.

A. Central and systemic hemodynamics: cardiac output, mean systemic arterial pressure (MSAP), central venous pressure (CVP), and systemic vascular pressure (SVR). (n = 6/group). tPA: tissue plasminogen activator; scuPA: single chain urokinase plasminogen activator; PFC: perfluorochemical.

| **Cardiac Out** | **PFC Only** | | **tPA 4 mg in PFC** | | **tPA 8 mg in PFC** | | **scuPA 4 mg in PFC** | | **scuPA 8 mg in PFC** | |
| --- | --- | --- | --- | --- | --- | --- | --- | --- | --- | --- |
| **(L/min)** | **mean** | **SEM** | **mean** | **SEM** | **mean** | **SEM** | **mean** | **SEM** | **mean** | **SEM** |
| Baseline | 5.2 | 0.2 | 5.9 | 0.4 | 5.9 | 0.5 | 5.8 | 0.5 | 6.2 | 0.3 |
| 3 hrs | 5.9* | 0.3 | 6.7* | 0.4 | 5.8 | 0.5 | 6.9* | 0.5 | 6.9* | 0.9 |
| 6 hrs | 6.3 | 0.5 | 6.4 | 0.5 | 5.7 | 0.5 | 6.6 | 0.6 | 6.2 | 0.3 |
| 12 hrs | 7.1 | 0.2 | 5.7†† | 0.3 | 5.6†† | 0.7 | 6.1† | 0.5 | 6.1†† | 0.4 |
| 18 hrs | 7.1 | 0.4 | 6.1† | 0.4 | 5.9†† | 0.9 | 5.9†† | 0.5 | 5.9†† | 0.4 |
| 24 hrs | 6.9 | 0.3 | 5.2†† | 0.3 | 5.4†† | 0.7 | 5.5†† | 0.3 | 5.7†† | 0.4 |
| 30 hrs | 7.4 | 0.4 | 4.9†† | 0.3 | 5.7† | 0.7 | 5.5†† | 0.3 | 5.9†† | 0.7 |
| 36 hrs | 7.9 | 0.4 | 5.7†† | 0.7 | 4.6†† | 0.3 | 6.2† | 0.3 | 6.2† | 0.6 |
| 42 hrs | 7.4 | 0.7 | 5.5†† | 0.3 | 4.5†† | 0.3 | 4.6†† | 0.4 | 6.1† | 0.4 |
| 48 hrs | 6.7 | 0.5 | 5.1†† | 0.4 | 4.8†† | 0.4 | 5.3†† | 0.6 | 6† | 0.5 |
|  |  |  |  |  |  |  |  |  |  |  |
| **MSAP** | **PFC Only** | | **tPA 4 mg in PFC** | | **tPA 8 mg in PFC** | | **scuPA 4 mg in PFC** | | **scuPA 8 mg in PFC** | |
| **(mm Hg)** | **mean** | **SEM** | **mean** | **SEM** | **mean** | **SEM** | **mean** | **SEM** | **mean** | **SEM** |
| Baseline | 93.5 | 4.8 | 97.5 | 3.0 | 95.8 | 3.3 | 94.7 | 5.2 | 96.5 | 3.0 |
| 3 hrs | 98* | 3.3 | 98.8 | 2.7 | 102.8* | 1.4 | 101.7* | 4.4 | 103.8* | 4.3 |
| 6 hrs | 100.2 | 3.1 | 98.7 | 3.7 | 97.2 | 3.1 | 97.3 | 5.7 | 98.0 | 2.5 |
| 12 hrs | 102.3 | 5.7 | 99.8 | 3.5 | 94.0 | 2.5 | 93.8 | 4.4 | 97.5 | 2.8 |
| 18 hrs | 92.7 | 4.2 | 94.8 | 1.7 | 92.0 | 2.6 | 95.7 | 2.6 | 96.8 | 2.4 |
| 24 hrs | 96.6 | 2.3 | 98.2 | 4.2 | 94.5 | 1.7 | 100.0 | 4.3 | 98.7 | 2.5 |
| 30 hrs | 94.0 | 4.8 | 95.2 | 2.0 | 96.0 | 2.9 | 97.2 | 4.6 | 100.8 | 4.7 |
| 36 hrs | 96.4 | 4.1 | 92.8 | 1.8 | 94.7 | 2.9 | 94.8 | 3.3 | 96.6 | 4.2 |
| 42 hrs | 95.8 | 5.8 | 98.7 | 3.3 | 94.3 | 3.4 | 95.2 | 3.4 | 96.0 | 2.0 |
| 48 hrs | 97.5 | 7.1 | 97.5 | 3.2 | 97.5 | 4.5 | 93.8 | 3.9 | 99.4 | 4.0 |
|  |  |  |  |  |  |  |  |  |  |  |
| **CVP** | **PFC Only** | | **tPA 4 mg in PFC** | | **tPA 8 mg in PFC** | | **scuPA 4 mg in PFC** | | **scuPA 8 mg in PFC** | |
| **(mm Hg)** | **mean** | **SEM** | **mean** | **SEM** | **mean** | **SEM** | **mean** | **SEM** | **mean** | **SEM** |
| Baseline | 4.7 | 0.9 | 4.3 | 0.7 | 4.3 | 0.9 | 4.3 | 0.9 | 5.8 | 0.7 |
| 3 hrs | 7.3* | 1.4 | 7.8* | 0.9 | 7.5* | 1.0 | 8.3** | 1.4 | 6.3* | 0.5 |
| 6 hrs | 6* | 1.4 | 6.5 | 0.7 | 7.3* | 0.7 | 5.7 | 0.8 | 4.7†† | 0.6 |
| 12 hrs | 6.7* | 1.1 | 4.5† | 1.0 | 5.8 | 0.5 | 6.0 | 0.4 | 5.8 | 1.0 |
| 18 hrs | 7.3* | 1.7 | 5.2† | 1.3 | 5.5†† | 0.4 | 5.8† | 0.7 | 6.2† | 1.6 |
| 24 hrs | 6.8* | 1.2 | 4.3† | 0.7 | 4.8†† | 0.8 | 6.2 | 0.5 | 5.2† | 1.0 |
| 30 hrs | 7.2* | 1.2 | 4.3† | 1.0 | 5.2†† | 0.7 | 6.8 | 1.2 | 5.8† | 1.0 |
| 36 hrs | 7.4* | 1.4 | 4.7† | 0.9 | 4.8†† | 0.7 | 5.4† | 0.9 | 6† | 1.1 |
| 42 hrs | 5.8 | 1.2 | 6.2 | 1.0 | 5.7 | 0.6 | 5.4 | 1.0 | 4.8† | 0.7 |
| 48 hrs | 8.3* | 2.9 | 5.2† | 1.0 | 5.7†† | 0.9 | 3.2†† | 1.0 | 6.2† | 1.1 |
|  |  |  |  |  |  |  |  |  |  |  |
| **SVR** | **PFC Only** | | **tPA 4 mg in PFC** | | **tPA 8 mg in PFC** | | **scuPA 4 mg in PFC** | | **scuPA 8 mg in PFC** | |
| **Woods Units** | **mean** | **SEM** | **mean** | **SEM** | **mean** | **SEM** | **mean** | **SEM** | **mean** | **SEM** |
| Baseline | 17.2 | 1.1 | 16.2 | 1.1 | 15.1 | 1.1 | 15.9 | 1.4 | 15.8 | 1.2 |
| 3 hrs | 15.5 | 1.1 | 14.9 | 1.0 | 15.1 | 2.1 | 13.7 | 0.9 | 17.8 | 2.6 |
| 6 hrs | 15.4 | 1.6 | 15.0 | 1.5 | 14.8 | 0.8 | 14.3 | 1.3 | 17.1 | 1.5 |
| 12 hrs | 13.6 | 0.8 | 17†† | 0.8 | 15.7† | 0.6 | 15.6† | 0.9 | 17.1†† | 1.2 |
| 18 hrs | 12.3 | 1.1 | 15.1† | 1.2 | 15† | 1.0 | 15.8† | 1.7 | 16.5†† | 1.7 |
| 24 hrs | 13.1 | 1.0 | 18.4†† | 1.4 | 16.3† | 1.1 | 17.4†† | 1.0 | 18.6†† | 2.2 |
| 30 hrs | 11.9 | 1.4 | 19†† | 1.3 | 16.4† | 1.6 | 16.5† | 0.6 | 17.8†† | 1.6 |
| 36 hrs | 11.4 | 0.4 | 16.8†† | 2.3 | 15† | 1.4 | 14.5† | 0.6 | 19.8††‡ | 0.9 |
| 42 hrs | 12.6 | 1.6 | 17.2† | 1.1 | 14.9 | 1.0 | 20.3† | 2.1 | 20.8† | 1.7 |
| 48 hrs | 13.8 | 2.4 | 18.4† | 1.2 | 15.7† | 1.0 | 18.2† | 2.2 | 19.7† | 0.9 |

*p < 0.05, **p < 0.01 vs. Baseline; †p < 0.05, ††p < 0.01 vs. PFC only; ‡p< 0.05, ‡‡p < 0.01 vs. Dose; ¥ p < 0.05; ¥¥ p < 0.01 vs. plasminogen activator type.

B. Pulmonary vascular hemodynamics: mean pulmonary artery pressure (MPAP), pulmonary capillary wedge pressure (PCWP), pulmonary vascular resistance (PVR). (n = 6/group). tPA: tissue plasminogen activator; scuPA: single chain urokinase plasminogen activator; PFC: perfluorochemical.

| **MPAP** | **PFC Only** | | **tPA 4 mg in PFC** | | **tPA 8 mg in PFC** | | **scuPA 4 mg in PFC** | | **scuPA 8 mg in PFC** | |
| --- | --- | --- | --- | --- | --- | --- | --- | --- | --- | --- |
| **(mm Hg)** | **mean** | **SEM** | **mean** | **SEM** | **mean** | **SEM** | **mean** | **SEM** | **mean** | **SEM** |
| Baseline | 17.0 | 1.0 | 17.0 | 0.7 | 18.2 | 0.5 | 17.3 | 0.9 | 17.8 | 0.9 |
| 3 hrs | 21* | 1.1 | 24.2* | 1.0 | 24.2* | 1.2 | 25* | 0.9 | 23.3* | 0.8 |
| 6 hrs | 24* | 1.2 | 27* | 1.6 | 21.7* | 0.8 | 23.3* | 0.8 | 24.5* | 0.8 |
| 12 hrs | 25.4* | 1.0 | 24.7* | 1.4 | 24* | 1.2 | 27.3** | 2.4 | 24.5* | 0.9 |
| 18 hrs | 25.7* | 1.9 | 24.2* | 1.9 | 24.7* | 1.8 | 26.2** | 0.9 | 21.7*¥ | 1.4 |
| 24 hrs | 25.8* | 2.0 | 24.2* | 2.1 | 30.3** | 3.3 | 26**¥ | 0.9 | 25*¥ | 1.0 |
| 30 hrs | 29.6** | 1.7 | 23.7* | 1.2 | 28* | 2.4 | 24.7* | 0.8 | 23.8*¥ | 1.5 |
| 36 hrs | 27* | 1.8 | 22.5* | 1.2 | 24.8* | 1.0 | 25.2* | 0.9 | 23.7* | 2.3 |
| 42 hrs | 23.3* | 1.8 | 23.7* | 1.8 | 25* | 1.7 | 22.4* | 1.2 | 22.8*¥ | 1.6 |
| 48 hrs | 25.8* | 1.9 | 24.3* | 0.8 | 29.6** | 2.4 | 22.4* | 1.4 | 24*¥ | 1.6 |
|  |  |  |  |  |  |  |  |  |  |  |
| **PCWP** | **PFC Only** | | **tPA 4 mg in PFC** | | **tPA 8 mg in PFC** | | **scuPA 4 mg in PFC** | | **scuPA 8 mg in PFC** | |
| **(mm Hg)** | **mean** | **SEM** | **mean** | **SEM** | **mean** | **SEM** | **mean** | **SEM** | **mean** | **SEM** |
| Baseline | 8.7 | 0.7 | 7.8 | 0.7 | 8.8 | 0.3 | 8.7 | 1.0 | 9.3 | 0.5 |
| 3 hrs | 11.7* | 1.1 | 14.8** | 0.8 | 13.2* | 0.9 | 15.7** | 1.1 | 14.3** | 0.6 |
| 6 hrs | 13.7* | 1.7 | 14.5** | 1.5 | 12.7* | 1.1 | 13* | 0.8 | 14.7** | 0.5 |
| 12 hrs | 13.6* | 1.0 | 12* | 0.9 | 13.5* | 1.0 | 13.3* | 1.0 | 14.2** | 0.6 |
| 18 hrs | 12.3* | 1.0 | 11.8* | 0.6 | 13.5* | 1.1 | 14.3* | 0.4 | 12.2*¥ | 0.7 |
| 24 hrs | 12.6* | 1.1 | 12* | 1.4 | 14.8** | 1.7 | 15.2** | 0.8 | 13.3*¥ | 0.7 |
| 30 hrs | 13.2* | 0.7 | 12.2* | 1.0 | 14.5** | 0.5 | 13.8* | 0.8 | 13.7*¥ | 0.8 |
| 36 hrs | 13.4* | 1.5 | 11.7* | 1.0 | 13* | 0.5 | 14.2** | 1.2 | 13.3* | 1.1 |
| 42 hrs | 13.3* | 0.9 | 12.3* | 1.3 | 11.4* | 0.9 | 11.4* | 0.5 | 12.2* | 0.5 |
| 48 hrs | 11* | 1.5 | 11.7* | 1.1 | 16.8** | 2.9 | 11* | 0.8 | 13.3*¥ | 1.7 |
|  |  |  |  |  |  |  |  |  |  |  |
| **PVR** | **PFC Only** | | **tPA 4 mg in PFC** | | **tPA 8 mg in PFC** | | **scuPA 4 mg in PFC** | | **scuPA 8 mg in PFC** | |
| **Woods Units** | **mean** | **SEM** | **mean** | **SEM** | **mean** | **SEM** | **mean** | **SEM** | **mean** | **SEM** |
| Baseline | 1.6 | 0.1 | 1.6 | 0.2 | 1.7 | 0.2 | 1.5 | 0.2 | 1.4 | 0.1 |
| 3 hrs | 1.6 | 0.1 | 1.4 | 0.2 | 2.0 | 0.3 | 1.4 | 0.2 | 1.4 | 0.1 |
| 6 hrs | 1.7 | 0.2 | 2.1* | 0.5 | 1.7 | 0.3 | 1.6 | 0.2 | 1.6 | 0.1 |
| 12 hrs | 1.7 | 0.2 | 2.3* | 0.2 | 2.0 | 0.2 | 2.4* | 0.4 | 1.7*‡ | 0.1 |
| 18 hrs | 1.9 | 0.2 | 2.1* | 0.4 | 2.1* | 0.3 | 2.1* | 0.3 | 1.7* | 0.2 |
| 24 hrs | 1.9* | 0.2 | 2.4* | 0.3 | 3.1* | 0.5 | 2* | 0.2 | 2.1*¥ | 0.1 |
| 30 hrs | 2.2* | 0.3 | 2.4* | 0.3 | 2.6* | 0.4 | 2* | 0.3 | 1.8*¥ | 0.3 |
| 36 hrs | 1.7 | 0.2 | 2.1* | 0.5 | 2.6* | 0.3 | 1.8 | 0.2 | 1.7*¥ | 0.3 |
| 42 hrs | 1.4 | 0.3 | 2.1* | 0.2 | 3.1* | 0.4 | 2.5* | 0.3 | 1.8*‡¥ | 0.2 |
| 48 hrs | 2.2* | 0.2 | 2.6* | 0.2 | 2.8* | 0.9 | 2.3* | 0.3 | 1.8*‡¥ | 0.2 |

*p < 0.05, **p < 0.01 vs. Baseline; †p < 0.05, ††p < 0.01 vs. PFC only; ‡p< 0.05, ‡‡p < 0.01 vs. Dose; ¥ p < 0.05; ¥¥ p < 0.01 vs. plasminogen activator type.
